# Stabilization of Cu Species in UiO‐66 Metal–Organic Framework for CO_2_‐to‐Methanol: Insights From Operando X‐ray and Electron Microscopy Studies

**DOI:** 10.1002/smll.74210

**Published:** 2026-07-01

**Authors:** Anna Liutkova, Fabio André Peixoto Esteves, Marianela López Romero, Margareth S. Baidun, Anastasia Molokova, Davide Salusso, Kirill A. Lomachenko, Andrea Testino, Alexander A. Kolganov, Evgeny A. Pidko, Emiliya Poghosyan, Marco Ranocchiari

**Affiliations:** ^1^ Center for Energy and Environmental Sciences PSI Villigen Switzerland; ^2^ Department of Chemistry and Applied Biosciences ETH Zürich Zurich Switzerland; ^3^ European Synchrotron Radiation Facility Grenoble France; ^4^ Inorganic Systems Engineering group Department of Chemical Engineering Faculty of Applied Sciences Delft University of Technology Delft The Netherlands; ^5^ ALBA synchrotron Barcelona Spain; ^6^ École Polytechnique Fédérale de Lausanne (EPFL) Lausanne Switzerland; ^7^ Center for Life Sciences PSI Villigen Switzerland

**Keywords:** CO_2_‐to‐methanol, density functional theory, electron microscopy, metal–organic frameworks, X‐ray absorption spectroscopy

## Abstract

UiO‐66, a Zr‐based metal–organic framework (MOF), provides an ordered porous environment capable of adsorbing/retaining CO_2_ and oxygenated intermediates relevant for methanol synthesis. Although UiO‐66‐based composites containing Cu and Cu‐Zn species show promising performance for CO_2_‐to‐methanol conversion, the role of the MOF framework in stabilizing the active metallic species under working conditions remains insufficiently understood. Here, the stabilization of copper species by UiO‐66 and the influence of Zn promotion on Cu speciation and catalytic performance during CO_2_ hydrogenation are investigated. Operando X‐ray absorption spectroscopy at the Cu, Zn, and Zr K‐edges is combined with electron microscopy and density functional theory calculations to follow the evolution of metal species and the framework during reduction, reaction, and transient switching experiments. Zn promotes the formation and stability of highly dispersed Cu under reaction conditions, while the Zr_6_ nodes of UiO‐66 respond dynamically through reversible hydroxylation and dehydroxylation. By correlating metal speciation, framework response, and methanol productivity under continuous‐flow conditions, this work provides molecular‐level insight into metal stabilization in Zr‐based MOFs and informs the design of MOF‐based catalysts for CO_2_ hydrogenation.

## Graphical abstract

This study reveals how a zirconium‐based metal–organic framework (UiO‐66) stabilizes copper species during CO_2_ hydrogenation to methanol. Operando X‐ray spectroscopy, electron microscopy, and theory show that zinc promotes highly dispersed copper and suppresses particle growth, while the framework dynamically responds to reaction intermediates. The findings guide the design of stable MOF‐based catalysts for carbon conversion.

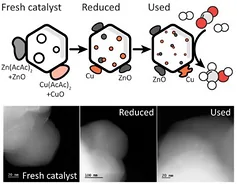

## 1 Introduction

Valorization of CO_2_ is important to mitigate global climate change and contribute to closing the carbon cycle [1]. In this scenario, researchers are searching for catalytic materials that combine high activity, selectivity, and stability under technologically relevant conditions [2, 3]. Recently, metal–organic frameworks (MOFs) attracted attention of researchers for valorization of CO_2_, including its capture and conversion [4, 5]. These materials combine organic linkers with inorganic nodes, offering well‐defined porosity and gas adsorption properties, while providing coordination environments that resemble those of metal‐oxide catalysts [6]. This integration enables stabilization of reaction intermediates and facilitates the formation of chemical bonds relevant for CO_2_ activation [7]. In addition, well‐defined metallic species can be incorporated into the MOFs [8], making them attractive systems for heterogeneous catalysis [9, 10], and operando spectroscopy allows the investigation of the metallic centers under reaction conditions [11, 12, 13]. Among Zr‐based MOFs, UiO‐66 is particularly attractive because of its exceptional thermal and chemical stability, originating from its Zr_6_ nodes connected by terephthalic acid linkers [14]. The Zr‐O and Zr‐OH functionalities can enhance CO_2_ binding and stabilize oxygenated intermediates [15, 16, 17, 18], while the ordered porosity of UiO‐66 provides confined environments for hosting metallic species active in CO_2_ hydrogenation. As an important function, UiO‐66 can accommodate structural defects, such as missing linker or missing cluster defects, without collapse of the framework owing to the high connectivity of the Zr_6_ nodes [19, 20]. These defects generate coordinatively unsaturated Zr‐O(H) sites and local mesoporosity when aggregated. This can act as anchoring point for introduced metal species and influence their dispersion, morphology, and stability under reaction conditions [21]. UiO‐66‐based catalysts for CO_2_‐to‐methanol conversion in continuous gas‐phase operation have been reported [22, 23], including several Cu‐ and Cu‐Zn‐containing UiO‐66 composites showing promising catalytic performance [22, 24, 25, 26]. Previous studies have addressed Cu stabilization in UiO‐66 using X‐ray absorption spectroscopy (XAS) [22] and density functional theory (DFT) calculations [27, 28, 29], suggesting that both confinement and active metal‐node interactions may play a role in the hydrogenation.

However, despite the growing number of reports, the role of the UiO‐66 framework in stabilizing the active metallic species under working conditions remains insufficiently understood. In particular, the interplay between active metal speciation and particle morphology has not been resolved. The role of the local coordination environment provided by Zr_6_ nodes and defects is also increasingly recognized as an important factor contributing to activity and stability in MOF‐based CO_2_ hydrogenation catalysts [22, 30, 31]. Distinguishing between confined metal clusters, interfacial metal‐oxide motifs, and external metal domains requires operando‐sensitive characterization, as the structure of the MOF‐based catalyst may evolve significantly during reduction and reaction [23].

In the present work, we investigate the role of the UiO‐66 framework and Zn promotion in stabilizing Cu species during CO_2_ hydrogenation to methanol. By combining operando X‐ray absorption spectroscopy at the Cu, Zn, and Zr edges with electron microscopy and density functional theory calculations, we directly correlate Cu and Cu‐Zn speciation, its particle morphology, and framework response under continuous‐flow reaction conditions, providing mechanistic insight into how UiO‐66 and its Zr‐O(H) environments contribute to the stabilization of catalytically relevant Cu species and enhanced methanol formation.

## 2 Results and Discussion

**FIGURE 1:**
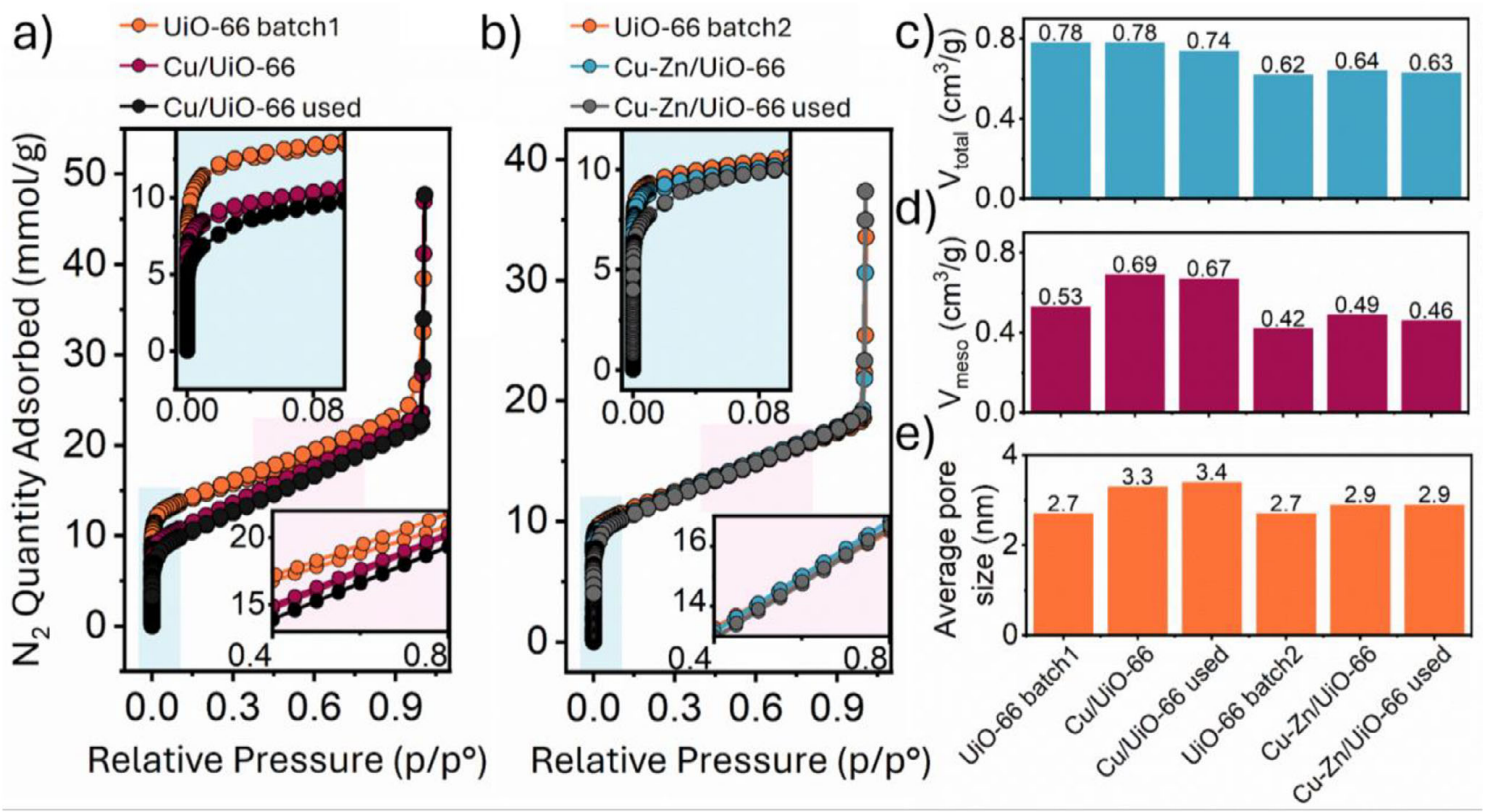
N_2_ physisorption isotherms of pristine UiO‐66 (two batches), fresh and used Cu/UiO‐66 and Cu‐Zn/UiO‐66 (a, b). Insets highlight the low‐ (blue) and intermediate‐pressure (pink) regions. Corresponding total pore volume (c), mesopore volume (d), and average pore size (e) derived from the adsorption data.

First, two batches of UiO‐66 were synthesized solvothermally following a reported procedure [30], yielding a phase‐pure, crystalline Zr‐based framework. We prepared Cu/UiO‐66 and Cu‐Zn/UiO‐66 with the same amount of Cu but promoted with Zn by wet impregnation of two batches of UiO‐66 using Cu or Cu and Zn acetylacetonate precursors under inert conditions. The synthesis is described in detail in the Experimental section. A scheme of the synthesis is provided in Figure S1. X‐ray diffraction measurements of the fresh catalysts demonstrate that the UiO‐66 framework is preserved after incorporation of the metallic species (Figure S2a,b). No additional peaks from phases of Cu are observed in both catalysts, only traces of ZnO in Cu‐Zn/UiO‐66, indicating that Cu and Cu‐Zn are introduced as highly dispersed species. ^1^H NMR spectroscopy further supports the presence of metallic precursors together with the UiO‐66 matrix (Figure S2c). Signals associated with acetylacetonate ligands and acetonitrile are detected for Cu‐ and Cu‐Zn‐loaded samples, whereas pristine UiO‐66 shows linker‐related resonances and residual dimethylformamide solvent from the MOF synthesis. These results indicate partial retention of precursor fragments after thermal treatment, consistent with metallic species being anchored within the porous framework rather than forming bulk phases. The textural properties of the materials were evaluated by N_2_ physisorption (Table 1, Figure 1). Each batch of pristine UiO‐66 exhibits a high surface area (*S_BET_* = 1150 and 920 m^2^ g^−1^) and total pore volume (*V_total_* = 0.78 and 0.62 cm^3^ g^−1^), however a difference in the surface area and pore volume may arise from differences in solvent exchange after the MOF synthesis. Upon Cu or Cu‐Zn incorporation, the surface area decreases to 940 and 880 m^2^ g^−1^ and the micropore volume to 0.09 and 0.15 cm^3^ g^−1^, while the average pore diameter increases slightly from 2.7 to 2.9 and 3.3 nm, respectively. Elemental composition determined by ICP‐OES demonstrates comparable Cu loadings of ca. 4 wt.% for both catalysts, with Cu‐Zn/UiO‐66 additionally containing ca. 10 wt.% Zn.

**TABLE 1:** N_2_ physisorption results and chemical composition of the UiO‐66‐based materials.

	*S_bet,_* (m^2^ g^−1^)	*S_micro,_* (m^2^ g^−1^)	*V_total_* (cm^3^ g^−1^)	*V_micro_*, (cm^3^ g^−1^)	*Av. Pore* *D_bet_* (nm)	Cu (wt.%)	Zn (wt.%)	Cu/Zr or (Cu+Zn)/Zr (wt.% / wt.%)
UiO‐66 batch 1	1150	560	0.78	0.25	2.7	—	—	—
Cu/UiO‐66 fresh	940	200	0.78	0.09	3.3	4.1	—	0.2
Cu/UiO‐66 used	890	160	0.74	0.07	3.4	3.7	—	0.1
UiO‐66 batch 2	920	460	0.62	0.20	2.7	—	—	—
Cu‐Zn/UiO‐66	880	320	0.64	0.15	2.9	4.1	10.2	0.4
Cu‐Zn/UiO‐66 used	870	370	0.63	0.17	2.9	4.6	11.0	0.4

Ex situ electron microscopy provides direct insight into the spatial distribution of Cu, Zn, and Zr species in the fresh catalysts. Pristine UiO‐66 consists of uniform, well‐defined particles with sizes in the 100–300 nm range and no contrast variations (Figures S3 and S5e,f). We did not notice substantial differences in morphology between the two batches. Both exhibit polycrystalline morphology with a certain degree of aggregation and crystal intergrowth, which is consistent with the presence of interparticle voids and mesopore volume contribution related to this. Fresh Cu/UiO‐66 shows a homogeneous distribution of Cu throughout the MOF particles, without the formation of distinct Cu domains (Figure S4a,b,e,f). In contrast, fresh Cu‐Zn/UiO‐66 exhibits dense regions attributed to Cu‐Zn‐rich domains, corroborated by Zn elemental mapping (Figure S4c,d,g,h). These domains are already present before the reduction, indicating that Zn influences the initial spatial distribution of introduced metallic species within the UiO‐66 framework. Overall, the combined XRD patterns, ^1^H NMR spectroscopy, N_2_ physisorption, ICP‐OES, and electron microscopy results demonstrate that UiO‐66 retains its crystallinity and porosity after Cu and Cu‐Zn species incorporation, while accommodating them as highly dispersed entities. These materials, therefore, provide a structurally well‐defined platform for probing the relationship between metal speciation, framework evolution, and catalytic performance in CO_2_ hydrogenation.

**FIGURE 2:**
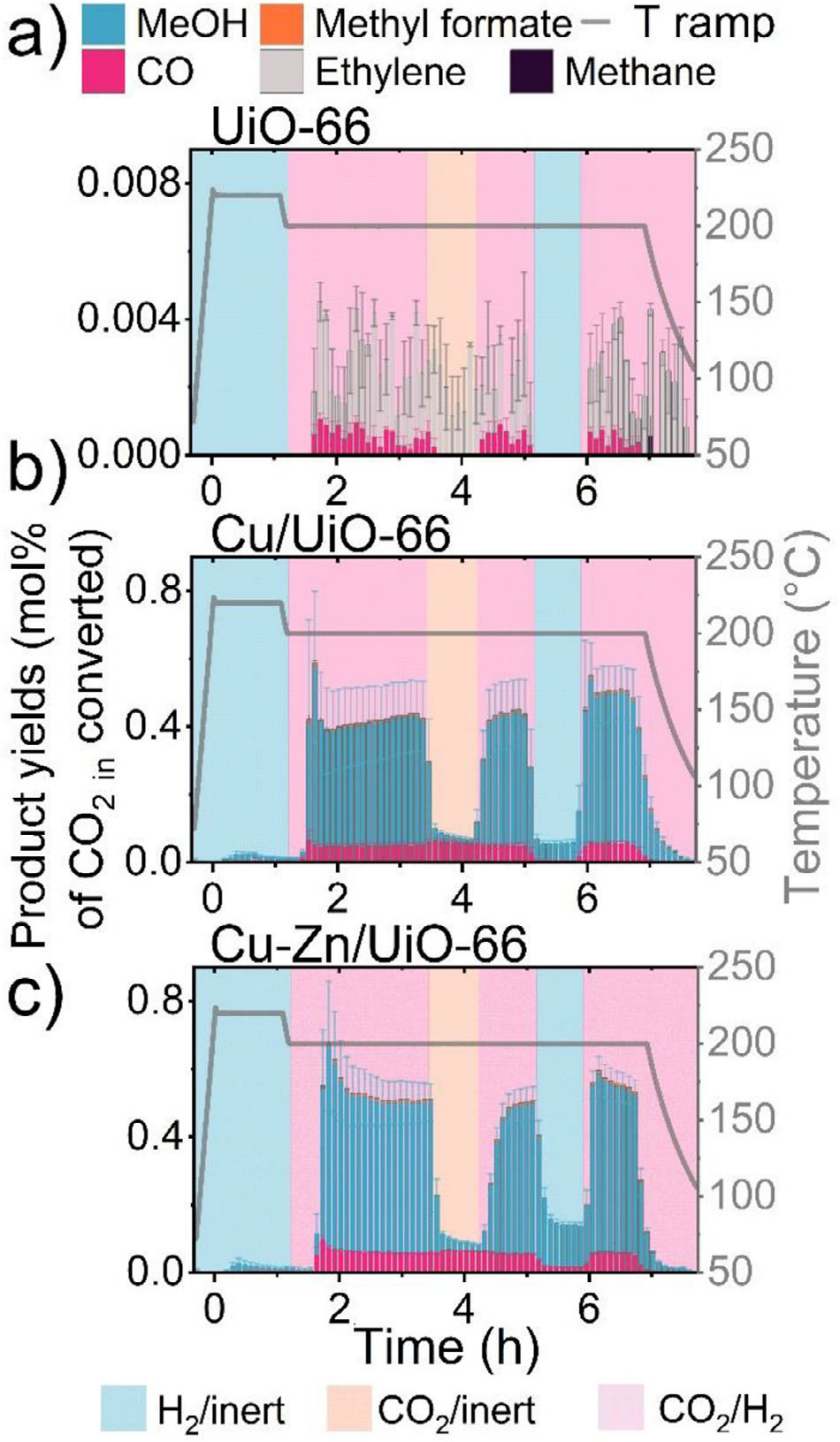
(a–c) UiO‐66, Cu/UiO‐66, and Cu‐Zn/UiO‐66 product yields. Conditions: reduction 220°C, 1 h, 5 bar 10% H_2_ in N_2_; reaction 200°C, 20 bar, CO_2_:H_2_ 1:3, PFR reactor, 50 mg catalyst + 100 mg SiC, 30 mL min^−1^ flow, GHSV 36 000 h^−1^.

**FIGURE 3:**
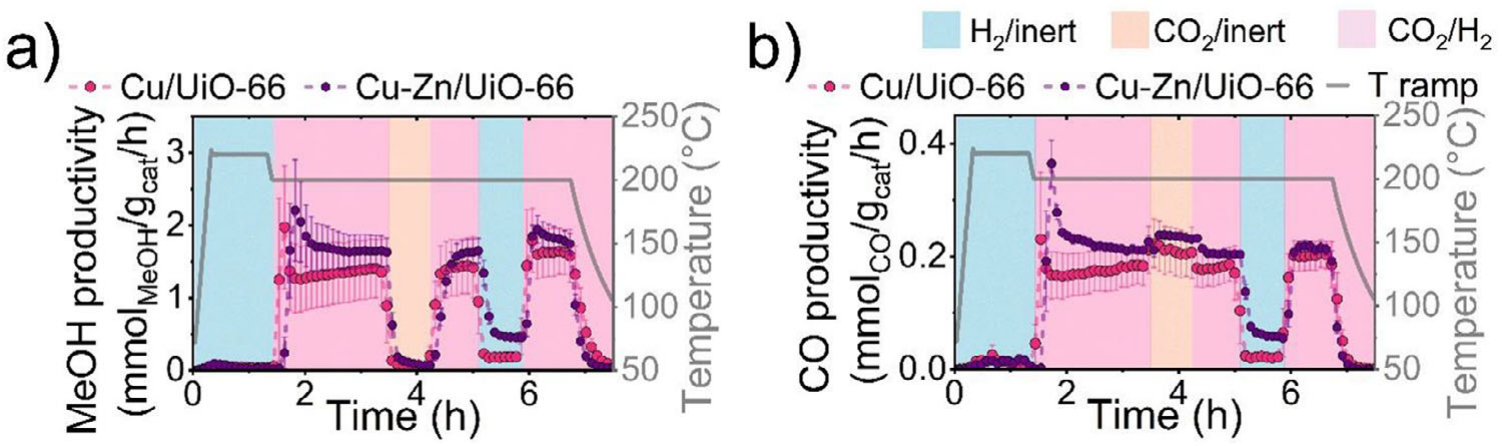
Cu/UiO‐66, and Cu‐Zn/UiO‐66 methanol productivity and CO productivity. Conditions: reduction 220°C, 1 h, 5 bar 10% H_2_ in N_2_; reaction 200°C, 20 bar, CO_2_:H_2_ 1:3, PFR reactor, 50 mg catalyst + 100 mg SiC, 30 mL min^−1^ flow, GHSV 36 000 h^−1^.

The catalytic performance of pristine UiO‐66, Cu/UiO‐66, and Cu‐Zn/UiO‐66 was evaluated in a continuous fixed‐bed reactor under identical reaction conditions (200°C, 20 bar, CO_2_:H_2_ = 1:3). The catalysts were activated prior to reaction by reduction in 10 vol.% H_2_ in N_2_ at 220°C and 5 bar total pressure, followed by cooling to reaction temperature and pressurization. Gas‐phase products were monitored online by gas chromatography, and subsequent transient experiments were performed by switching the feed composition during steady‐state operation (Figures 2 and 3). Pristine UiO‐66 shows little/no catalytic activity toward methanol or CO formation under the applied conditions. Small amounts of oxygenated species were initially detected and attributed to desorption of residual synthesis‐related compounds rather than catalytic conversion. Apart from trace signals, no hydrocarbons or oxygenates were produced over UiO‐66, demonstrating that the bare framework is catalytically inactive for CO_2_ hydrogenation (Figure 2a). In contrast, Cu/UiO‐66 exhibits significant activity toward methanol formation. Under steady‐state CO_2_ hydrogenation, methanol is the dominant product with a selectivity of approximately 90%, accompanied by minor CO formation (Figures 2b and 3b).

The average methanol productivity for Cu/UiO‐66 reaches ca. 1.8 mmol MeOH g_cat_^−1^ h^−1^, while CO productivity is about one order of magnitude lower (Figure 3). These results demonstrate that Cu species introduced into the UiO‐66 framework are catalytically active for CO_2_ hydrogenation to methanol.

Further enhancement is observed upon Zn promotion. Cu‐Zn/UiO‐66 displays higher methanol productivity than Cu/UiO‐66 at identical Cu loading, reaching ca. 2.1 mmol MeOH g _cat_^−1^ h^−1^ on average (or 44 and 51 mmol g _Cu_^−1^ h^−1^, respectively; Figures 2c and 3a). The methanol selectivity remains close to 90%, indicating that Zn primarily affects activity rather than selectivity. Overall, the bimetallic Cu‐Zn/UiO‐66 catalyst is approximately 15%–20% more active than the monometallic Cu/UiO‐66 under the same reaction conditions. At this stage, this enhancement indicates a Zn promotional effect, while its structural origin cannot be assigned from catalytic data alone. Such promotional effects are well established in Cu‐Zn systems, where Zn‐derived species enhance Cu dispersion and catalytic performance [32, 33]. We also note that the presence of additional mesoporous volume may influence transport phenomena. Literature data for Cu/UiO‐66 systems with comparable Cu loadings but markedly different textural properties show similar orders of magnitude in methanol yields (Table S1), indicating that the catalytic trends discussed here cannot be attributed solely to porosity effects.

Transient experiments within the same experimental protocol provide additional insight into the role of the MOF host and the Cu and Cu‐Zn species. When the H_2_ feed is switched off while maintaining CO_2_ flow, methanol formation decreases rapidly over both catalysts, indicating that gas‐phase hydrogen is required for continuous methanol formation. However, activity to CO can be maintained by the possible reaction of CO_2_ with the remaining hydrogenated species in the MOF pores. Conversely, when CO_2_ is switched off, and H_2_ is maintained, methanol and CO signals decay both, and more slowly. This delayed depletion indicates that the MOF itself can retain CO_2_, temporarily available for hydrogenation [15, 18, 34]. The effect is more pronounced for Cu‐Zn/UiO‐66, possibly because of enhanced interaction of CO_2_ with the Zn species [35, 36]. After a switch back to the reaction mixture, an increase in the methanol and CO production for both materials is observed because of possible Cu redispersion, indicating that the dispersion of Cu is an important factor affecting catalytic activity in CO_2_ hydrogenation [37].

**FIGURE 4:**
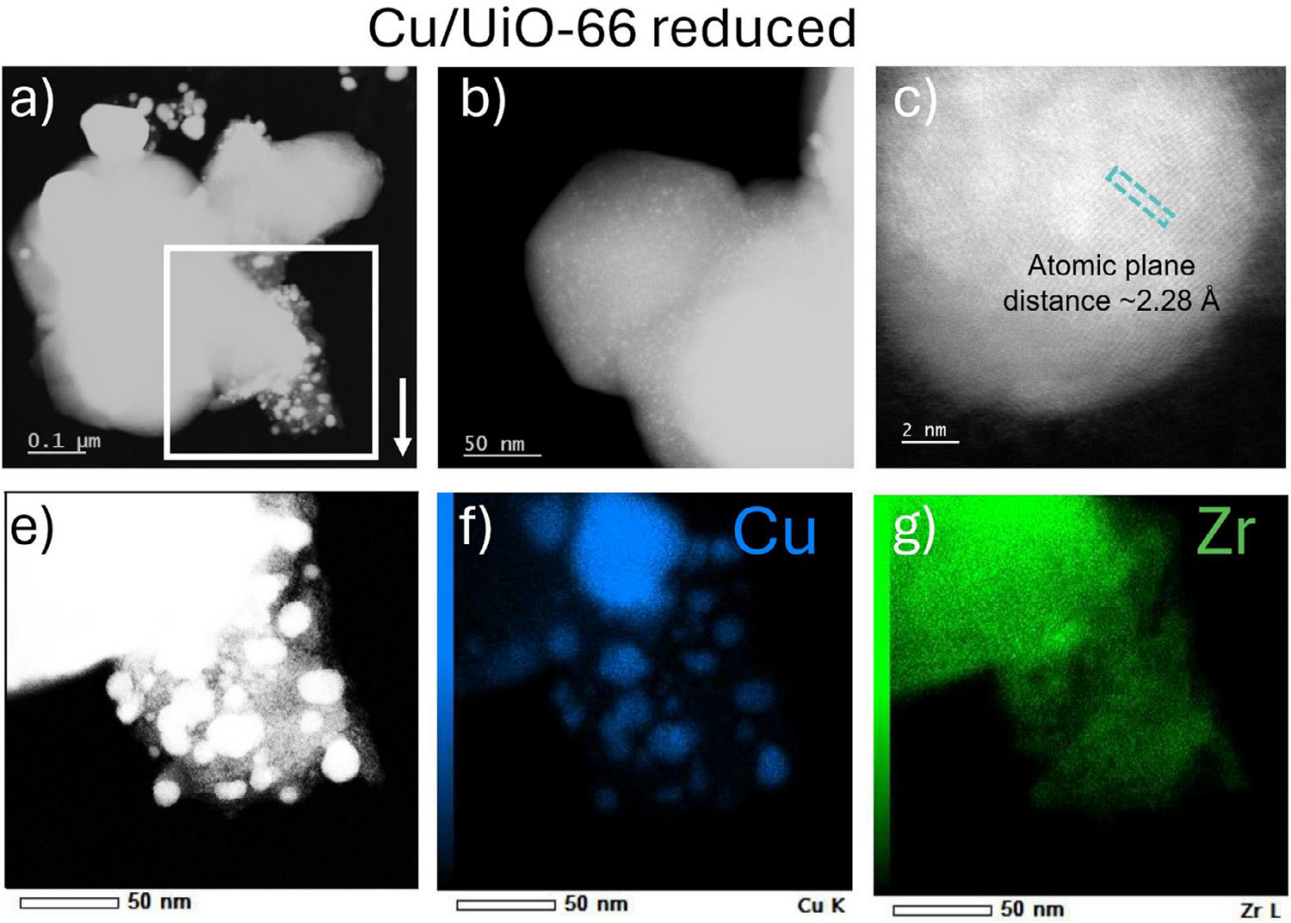
HAADF‐STEM micrographs of reduced Cu/UiO‐66 samples show the presence of Cu species with varying size distributions (a,b). Larger Cu particles exhibit a crystalline structure (c). EDX‐based elemental mapping (e–g) demonstrates the presence and spatial distribution of the Cu species.

Electron microscopy was used to study the morphology of the UiO‐66 framework and the spatial distribution of Cu and Cu‐Zn species in the reduced and used catalysts. After reduction, significant changes in the morphology of the introduced metallic species are observed. In reduced Cu/UiO‐66, Cu‐based particles with a broad size distribution are present within the MOF crystals, ranging from approximately 2 to 30 nm (Figure 4a,b).

**FIGURE 5:**
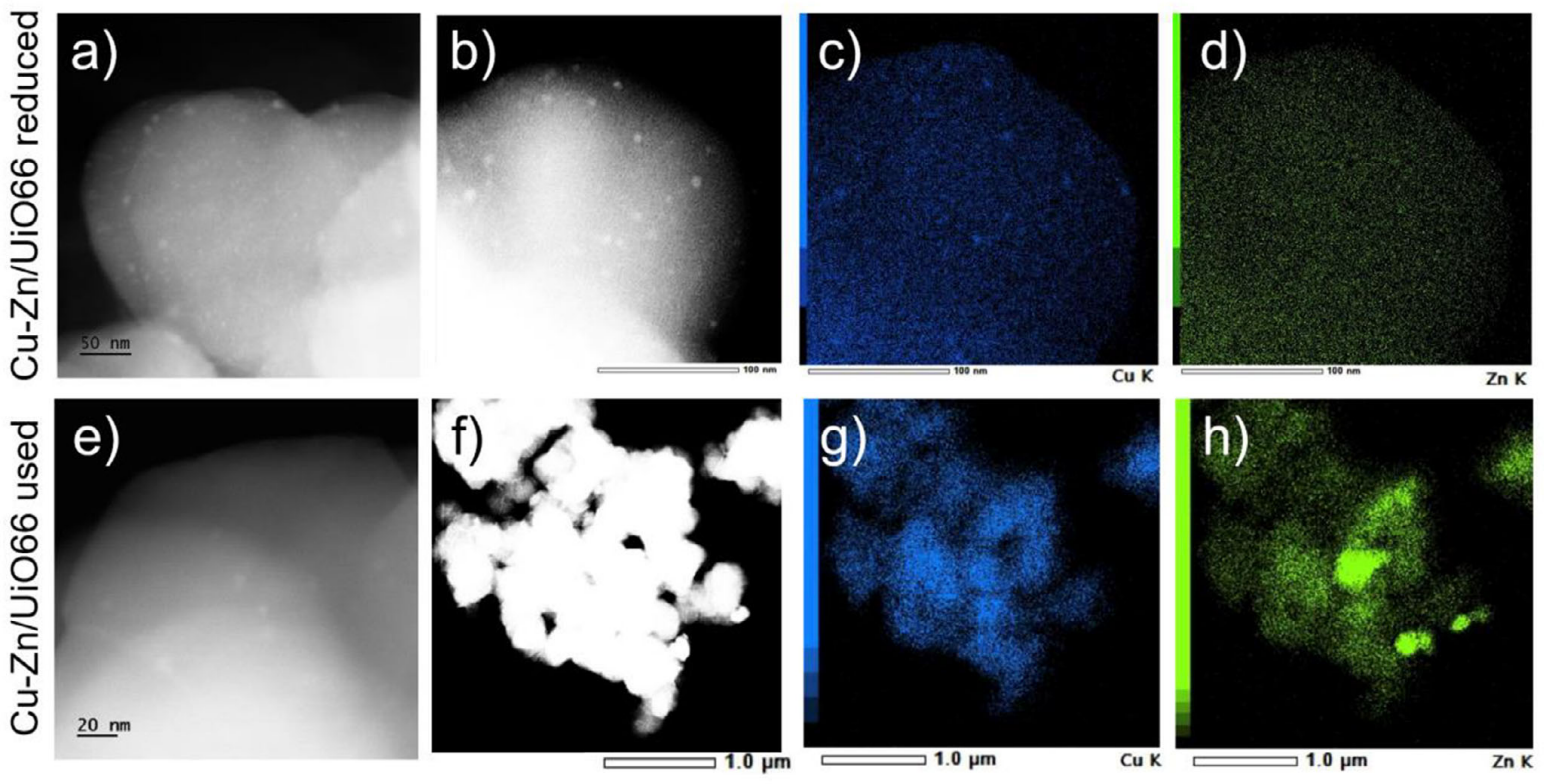
HAADF STEM micrographs of reduced (a) and used (e) Cu‐Zn/UiO‐66 showing small bright nanoparticles decorating the MOFs. EDX‐based elemental maps (b–d, f–h) demonstrate the presence and distribution of Cu and Zn species for both reduced and used catalysts.

High‐resolution TEM reveals that the larger Cu particles are crystalline, as demonstrated by the presence of lattice fringes (Figure 4c and Figure S5d). STEM‐EDX elemental mapping shows that these Cu particles are retained within the MOF matrix and spatially correlated with the Zr‐containing framework (Figure 4e–g). These observations indicate that Cu species coalesce during reduction while remaining confined within the UiO‐66 crystals. In contrast, reduced Cu‐Zn/UiO‐66 exhibits a different metal distribution. HAADF‐STEM images show smaller bright nanoparticles decorating the MOF particles (Figure 5a). Elemental mapping demonstrates the presence and colocalization of Cu and Zn species within the framework (Figure 5c,d).

In addition to the Cu‐rich species, larger Zn‐containing crystalline domains are observed, often located near the edges of the MOF crystals in Cu‐Zn/UiO‐66. Line‐scan analysis and high‐resolution HAADF‐STEM imaging reveal the crystalline nature of these domains, with lattice fringes of approximately 2.62 Å consistent with ZnO‐like domains (Figure S5a,c). The morphology of Cu‐Zn/UiO‐66 remains similar after catalytic operation. Used Cu‐Zn/UiO‐66 shows small Cu‐rich species retained within the MOF together with larger Zn‐containing crystalline domains comparable to those observed in the reduced state (Figure 5e–h). STEM‐EDX elemental maps of the used catalyst demonstrate that both Cu and ZnO remain present and spatially distributed in a manner similar to the reduced sample, indicating that the metal distribution established during reduction persists under reaction conditions. No additional Cu‐ or Cu‐Zn‐containing crystalline phases are detected by XRD in the reduced and used Cu‐Zn/UiO‐66 (Figure S10), indicating that Zn is present predominantly as ZnO domains. STEM‐EDX mapping further demonstrates co‐localization of Zn, Cu, and O within the same regions, supporting spatial association between Zn‐containing oxide domains and Cu‐rich species (Figure S6).

We estimated the size of the Cu particles for the reduced Cu/UiO‐66 and reduced/used Cu‐Zn/UiO‐66 from several images. The sizes of 5 ± 0.4 and 4 ± 0.3 nm were estimated for reduced and used Cu‐Zn/UiO‐66 (averaged from 12 particles and 10 particles, accordingly), and 13 ± 1.5 nm for reduced Cu/UiO‐66 (16 particles counted). This shift toward smaller Cu‐rich species in Cu‐Zn/UiO‐66 suggests that the higher methanol productivity may partly arise from increased Cu dispersion and accessible Cu surface area [38, 39]. We notice that particle size statistics are limited by the high Zr contrast of the UiO‐66 matrix, which limits reliable identification of small Cu‐rich domains. Additional low‐ and high‐magnification images supporting the particle‐size estimation are provided in Figures S7–S9. Taken together, electron microscopy demonstrates that Cu species in Cu/UiO‐66 undergo significant coarsening during reduction, leading to a wide particle size distribution spatially associated with the UiO‐66 crystallites (e.g., at defects or internal interfaces). In contrast, Cu‐Zn/UiO‐66 is characterized by small Cu‐rich species and distinct ZnO‐rich crystalline domains, which are already present in the fresh catalyst and remain stable after reduction and catalytic tests.

To support the EM findings on the redispersion of introduced metallic species, we carried out the N_2_ physisorption measurements after the catalytic tests (Figure 1). Both Cu/UiO‐66 and Cu‐Zn/UiO‐66 show a further reduction in micropore volume, accompanied by an increase in mesopore volume contribution. This evolution is attributed to initial micropore blocking by introduced Cu and Cu‐Zn species. Later, this was followed by their migration and redispersion during reduction and reaction, resulting in partial recovery of micropores for used Cu‐Zn/UiO‐66 and an increase of the average pore size for used Cu/UiO‐66. The observation on structural changes for the pristine UiO‐66 and MOF‐based catalysts before and after catalysis is also supported by the XRD of the used catalysts with the peaks of ZnO preserved within the MOF and no new phases present (Figure S10). Minor changes in the local environment of the Zr_6_O_4_(OH)_4_ nodes can be observed from the ex situ Raman spectroscopy (Figure S11). In the 600–700 cm^−1^ region, the *ν*(Zr‐O) band around ∼630 cm^−1^ becomes broader after catalysis for the Cu‐ and Cu‐Zn‐modified samples, and an overall broadening of Raman features and an increase of background intensity is observed for the used materials [40]. The latter can be explained because of a buildup of retained reaction intermediates [41], since the crystallinity of the MOF is preserved after the catalysis. The ∼1430 cm^−1^ signal (*νs*(O‐C‐O)) demonstrates splitting, which appears as a doublet in pristine UiO‐66. In the fresh Cu‐Zn/UiO‐66 this feature becomes a single band, while after catalysis, the doublet reappears, indicating a change associated with Zn. In contrast, for Cu/UiO‐66 the doublet is present in both fresh and used materials. Later, we attempted to quantify the amount of possibly missing linkers for the pristine materials from thermogravimetric analysis coupled with mass spectrometry (TGA‐MS, Figure S12) [42, 43]. The results indicated a stoichiometrically correct composition of the pristine MOF or slightly more organics present due to residual dimethylformamide from the MOF synthesis (Figure S2c). Also, the possible presence of the missing nodes in UiO‐66 can be deduced from the “forbidden” XRD reflections [31] (Figure S10a).

**FIGURE 6:**
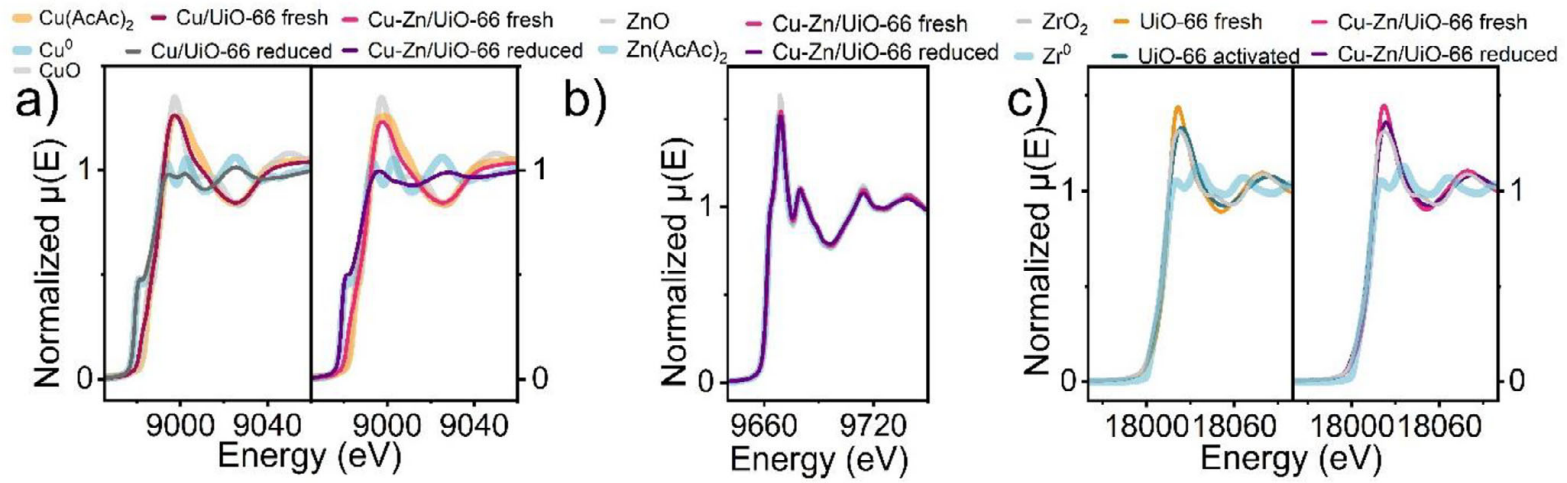
Operando XANES at the ROCK beamline (SOLEIL) during CO_2_ hydrogenation at 200°C, 20 bar, 30 mL min^−1^, CO_2_:H_2_ = 1:3. Cu K‐edge XANES for fresh/reduced Cu/UiO‐66 and Cu‐Zn/UiO‐66 compared to reference spectra (a), Zn K‐edge XANES for fresh/reduced Cu‐Zn/UiO‐66 (b), Zr K‐edge for fresh/activated UiO‐66 and fresh/reduced Cu‐Zn/UiO‐66 compared to reference spectra (c).

Operando X‐ray absorption spectroscopy was employed to resolve the speciation of Cu, Zn, and Zr under reduction, reaction, and transient switching conditions. Measurements were carried out at the ROCK beamline using an “edge‐jumping” [44] setup that enables the simultaneous acquisition of Cu, Zn, and Zr K‐edge spectra by alternating between two detector cameras tuned to different energies. The schematic of the experimental configuration and a photograph of the experimental stage are provided in Figures S13 and S14. The applied temperature, pressure, gas composition, and switching protocol were identical to those used during catalytic testing, enabling direct comparison between catalytic performance and spectroscopic observations. The MS signals, related to the gas mixtures and production of methanol, are provided in Figure S15. From a primary estimate, the Cu K‐edge exhibits the most pronounced changes among the three elements investigated. X‐ray absorption near edge structure (XANES) spectra of the fresh Cu/UiO‐66 and Cu‐Zn/UiO‐66 catalysts show similar features consistent with a mixture of Cu precursor species prior to reduction (Figure 6a).

**FIGURE 7:**
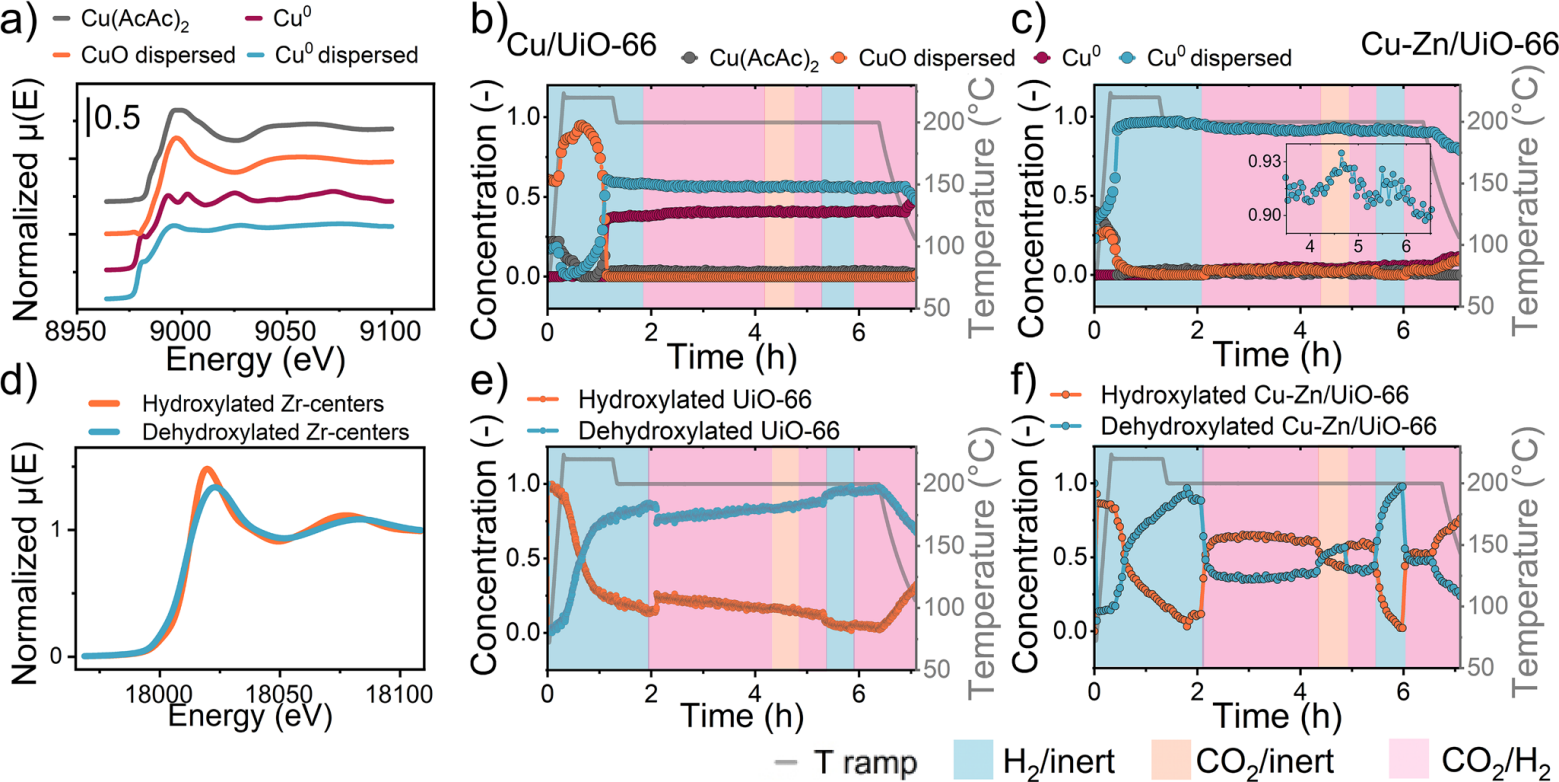
Operando XANES at the ROCK beamline (SOLEIL) during CO_2_ hydrogenation at 200°C, 20 bar, 30 mL min^−1^, CO_2_:H_2_ = 1:3. Cu K‐edge MCR‐ALS reference/component spectra (Cu(acac)_2_, CuO [48] dispersed, higher‐coordination metallic Cu (Cu^0^), Cu^0^‐dispersed species [46, 47]) (a) and corresponding concentration profiles for Cu/UiO‐66 (b) and Cu‐Zn/UiO‐66 (c). Enlarged inset in (d) – evolution of the Cu^0^‐dispersed upon the switches. Zr K‐edge MCR‐ALS component spectra assigned to hydroxylated and dehydroxylated Zr centers (e) and corresponding concentration profiles for UiO‐66 (h) and Cu‐Zn/UiO‐66 (f).

To follow the changes observed at Cu K‐edge, we used multivariate‐curve resolution‐alternating least squares (MCR‐ALS) analysis, on both combined datasets Cu/UiO‐66 and Cu‐Zn/UiO‐66. MCR‐ALS was performed using four components. Components Cu(AcAc)_2_ and Cu^0^ were taken from experimental spectra of the precursor (Cu(AcAc)_2_) and Cu foil (Cu^0^). Other two components were initialized using SIMPLISMA [45] and were allowed to evolve during ALS optimization. The component assigned as Cu^0^‐dispersed is in good agreement with literature data for metallic Cu nanoparticles [46, 47]. The intermediate CuO‐dispersed component is in agreement with the spectra of CuO nanoparticles [48, 49]. MCR‐ALS analysis of the Cu K‐edge dataset identifies contributions from CuO dispersed and Cu(acac)_2_‐derived species in both fresh materials (Figure 7a–c). Upon reduction, Cu/UiO‐66 evolves toward a mixed Cu state. MCR‐ALS reveals first, the evolution of the remaining precursor toward dispersed CuO [48], and then the formation of both higher‐coordination metallic Cu and a dispersed Cu component [46, 47]. The higher‐coordination Cu contribution forms during the reduction step and remains significant under reaction conditions, not affected by flow switching (Figure 7b). Derived from the Cu foil reference spectrum, this component reflects Cu environments spectroscopically similar to higher‐coordination metallic Cu rather than formation of a Cu foil phase. Since XANES features reflect a combination of coordination, oxidation state, and disorder, the extracted components are interpreted in terms of relative coordination environments and likely contain contributions from metallic Cu species with different particle sizes and degrees of dispersion.

The evolution of copper is consistent with the electron microscopy observations showing a broad Cu particle size distribution after reduction (Figure 4). In contrast, Cu‐Zn/UiO‐66 shows a distinct Cu evolution pathway. During reduction, Cu species rapidly evolve toward a dispersed Cu^0^ component, referred to in the MCR‐ALS analysis as very small Cu species [46, 47] (Figure 7c). This dispersed Cu^0^ state remains dominant during CO_2_ hydrogenation. Small but detectable changes in the Cu^0^‐dispersed component fractions are observed during feed switches, indicating that the surface of the active Cu is higher than for Cu/UiO‐66 (Figure 7c inset, Figure S16). However, the overall Cu speciation remains largely preserved under the high‐pressure hydrogenation conditions. During the cooldown, the system was kept under the reaction mixture. At decreasing temperature, H_2_‐driven reduction becomes kinetically limited, while mild oxidation associated with CO_2_ and residual reaction water dominates, which can explain the increase of CuO‐like contribution and decrease of the higher‐coordination metallic Cu fraction for both catalysts. The references, spectra for analysis used, and corresponding residuals are provided in Figures S17–S19.

In contrast to Cu, Zn K‐edge XANES spectra show no significant changes during reduction, reaction, or gas switching over Cu‐Zn/UiO‐66 (Figure 6b and Figure S20). The spectral features remain unchanged within the experimental protocol, indicating that Zn remains spectroscopically inert under the applied conditions; however, this does not imply that Zn is catalytically inert. The Zn K‐edge XANES spectra are consistent with a ZnO‐like local environment. ZnO‐like species represent the dominant Zn‐containing phase in the material, as supported by electron microscopy and XRD. This was corroborated by comparison of Cu‐Zn/UiO‐66 XANES spectrum with a ZnO reference spectrum (Figure S21). Furthermore, fitting of extended X‐ray absorption fine structure (EXAFS) spectra using the ZnO structural model provides a good description of the data and confirms the preservation of the ZnO coordination environment under reaction conditions (Figures S34 and S35, Table S2). Comparison with a Cu‐Zn alloy (brass) reference shows no spectral similarity, indicating that alloy formation is not observed. While Cu‐Zn interfacial sites cannot be excluded, they are not directly detected by the present measurements and are only suggested by the computational analysis (vide infra). In this context, Zn is interpreted to act primarily as a structural promoter that stabilizes dispersed Cu species, while its direct participation in the catalytic cycle cannot be resolved from the present data. Because no meaningful spectral evolution was observed, MCR‐ALS analysis was not applied to the Zn K‐edge dataset.

The Zr K‐edge XANES provides information on the response of the UiO‐66 framework during activation and reaction (Figures 6c and 7d). For pristine UiO‐66, the Zr spectra show a gradual evolution from a hydroxylated to a dehydroxylated Zr environment [34] during the activation procedure (Figure 7e). No significant response to gas composition switches is observed, consistent with the absence of catalytic activity for CO_2_ hydrogenation. For Cu/UiO‐66 and Cu‐Zn/UiO‐66, the Zr environment evolves differently. During pretreatment, the Zr nodes transition toward a dehydroxylated state (Figure 7d,f and Figure S22). Under CO_2_ hydrogenation conditions, the Zr centers become partially hydroxylated again, consistent with the formation of oxygenated reaction products and intermediates, including methanol and water [34]. We note that these changes reflect the response of the Zr environment to adsorbed species under reaction conditions and do not establish a direct role of Zr nodes in the catalytic cycle, but rather reflect adsorption of reaction intermediates and products. Gas switching induces further changes in the Zr K‐edge spectra. In the absence of H_2_, the Zr environment shifts toward a partially dehydroxylated state, where CO_2_ can react with the remaining hydrogenated intermediates in the MOF pores, as we see from the catalytic performance data. In the absence of CO_2_, the Zr centers become quickly dehydroxylated again, indicating elution of the CO_2_ hydrogenation intermediates and products from the MOF pores. During the cooldown in reaction mix, the increase of the hydroxylated Zr fraction is consistent with stronger adsorption of polar molecules as CO_2_ and residual reaction water at lower temperature. The reversible nature of these changes can be observed in the time‐resolved Zr XANES data (Figure S23), with residuals of the MCR‐ALS analysis provided in Figure S24. Thus, the Zr K‐edge XANES results indicate a dynamic response of the framework to the reaction environment, while a direct contribution of Zr nodes to catalytic turnover cannot be concluded from the present data. Taken together, the operando XANES results show that Cu speciation is strongly affected by Zn promotion, with Cu‐Zn/UiO‐66 stabilizing dispersed Cu species during reduction and reaction. Zn remains spectroscopically inert under the given conditions, while the Zr nodes of UiO‐66 respond dynamically to the presence of oxygenated species, undergoing reversible hydroxylation and dehydroxylation during reaction and gas switching.

**FIGURE 8:**
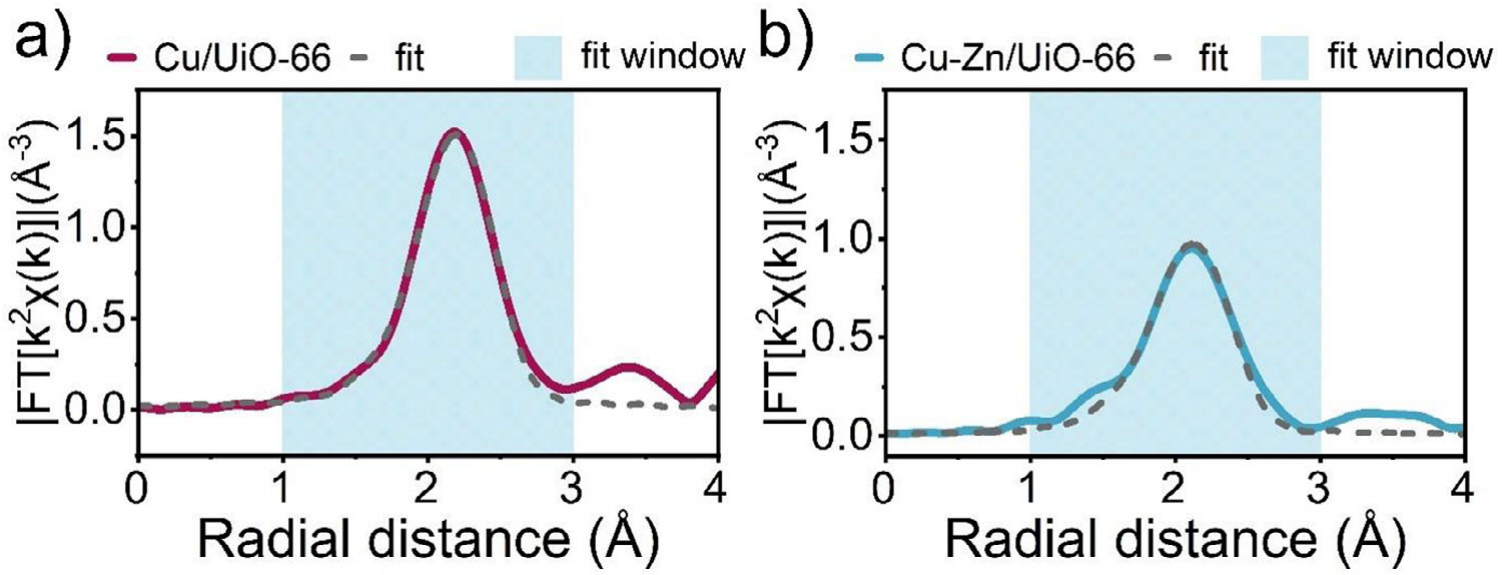
|FT| of averaged k^2^‐weighted EXAFS spectra and their fit of the dispersed metallic Cu species in Cu‐Zn/UiO‐66 and Cu/UiO66 samples. Experimental and best fit curves of imaginary components and EXAFS spectra are reported in Figure S33.

Cu K‐edge EXAFS was analyzed to support the Cu speciation trends derived from XANES. Representative EXAFS spectra with fits are shown in Figure 8, while raw data, additional fit outputs, and fitting details are provided in Figures S26, S27, S32, and S33.

The fitted structural parameters are summarized in Table 2. The EXAFS data for reduced catalysts are dominated by Cu‐Cu scattering contributions, with estimated first‐shell coordination numbers (*CN*) of 9.2 for Cu/UiO‐66 and 6.2 for Cu‐Zn/UiO‐66. In the fitted models shown in Figure 8, the first‐shell Cu‐Cu contribution reproduces the main peak in R‐space, and the fitted coordination numbers are lower than those expected for higher‐coordination metallic Cu (*CN* = 12, Figure S32).

**TABLE 2:** Fit results of the averaged spectra of the reduced Cu species in Cu/UiO‐66 and Cu‐Zn/UiO‐66. Details on the EXAFS fit analysis can be found in Section S8.

Sample	Path	*N*	*S_0_^2^*	*N_av_*	*σ^2^* (Å^2^)	*E_0_* (eV)	*R* (Å)	*R‐*factor
Cu/UiO‐66	Cu‐Cu	12	0.9 (fixed)	9.2 ± 0.7	0.014 ± 0.0008	3.2	2.52 ± 0.006	0.007
Cu‐Zn/UiO‐66	Cu‐Cu	12	0.9 (fixed)	6.2 ± 0.9	0.015 ± 0.002	−0.005	2.47 ± 0.011	0.022
Cu foil	Cu‐Cu	12	0.90 ± 0.05 (fitted)	12 (fixed)	0.0088 ± 0.0005	4.129	2.54 ± 0.003	0.003

In this context, although coordination number and Debye–Waller factors correlate, leading to an imprecise evaluation of both, the lower values observed for Cu‐Zn/UiO‐66 (*CN* = 6.2; *R* = 2.47 Å) closely match reported values for activated Cu/UiO‐66 systems with dispersed Cu species [22]. The higher coordination number obtained for Cu/UiO‐66 (*CN* = 9.2; R = 2.52 Å) indicates a shift toward higher average Cu‐Cu coordination, consistent with larger Cu domains. Together with the XANES/MCR‐ALS analysis, these coordination numbers are consistent with a larger fraction of higher‐coordination Cu environments in Cu/UiO‐66 compared to Cu‐Zn/UiO‐66. The CN value obtained for Cu/UiO‐66 is furthermore consistent with the coexistence of higher‐ and lower‐coordination Cu environments observed by XANES/MCR‐ALS analysis, although EXAFS alone cannot uniquely distinguish this situation from a distribution of intermediate‐sized Cu domains. The EXAFS‐derived coordination numbers independently support the XANES/MCR‐ALS analysis, with Cu‐Zn/UiO‐66 dominated by lower‐coordination Cu environments and Cu/UiO‐66 showing mixed higher‐ and lower‐coordination Cu contributions. Comparable coordination numbers and Cu‐Cu distances have also been reported for small metallic Cu clusters on oxide supports under reaction‐relevant conditions [47]. Using established EXAFS‐based coordination number estimates for metallic nanoparticles [50], the coordination numbers observed here are consistent with Cu clusters in the ∼1 nm size regime for Cu‐Zn/UiO‐66, supporting the use of small MOF‐confined Cu and Cu‐Zn cluster models in the DFT analysis. Furthermore, the density functional theory (DFT)‐calculated radial distribution functions (RDFs) for MOF‐confined nanoparticles (vide infra) demonstrate structural disorder, which increases with the amount of Zn incorporated into the nanoparticles (Figure S38). This distribution of Cu‐Cu distances provides a natural explanation for the comparatively high Debye–Waller factors observed in the EXAFS analysis (*σ^2^* = 0.014–0.015 Å^2^), reflecting structural disorder associated with small and dispersed Cu domains.

The DFT calculations were performed to rationalize the stabilization of Cu and Cu‐Zn species in UiO‐66 observed experimentally by XANES, EXAFS, and electron microscopy. Although the experimental system exhibits substantial structural complexity, including different defect types and distribution of nanoparticle sizes, explicitly capturing the full complexity within a single atomistic model is computationally prohibitive. The experimentally observed low coordination numbers and dispersed Cu environments motivated the use of small MOF‐confined Cu and Cu‐Zn cluster models in the DFT analysis. We therefore employ a deliberately simplified and well‐defined model that enables systematic comparison of stabilization trends while being grounded in a plausible experimental scenario. The chosen model consists of a defective UiO‐66 framework containing a missing‐linker defect, created by removing one terephthalate linker, with small Cu or Cu‐Zn clusters confined within the defect cavity. Other experimentally plausible defect scenarios, such as missing‐node defects that could host larger nanoparticles, are not considered due to their substantially higher computational cost. Accordingly, the present model is intended to rationalize relative stability trends rather than to reproduce the full experimental structural complexity or uniquely identify the dominant defect character.

**FIGURE 9:**
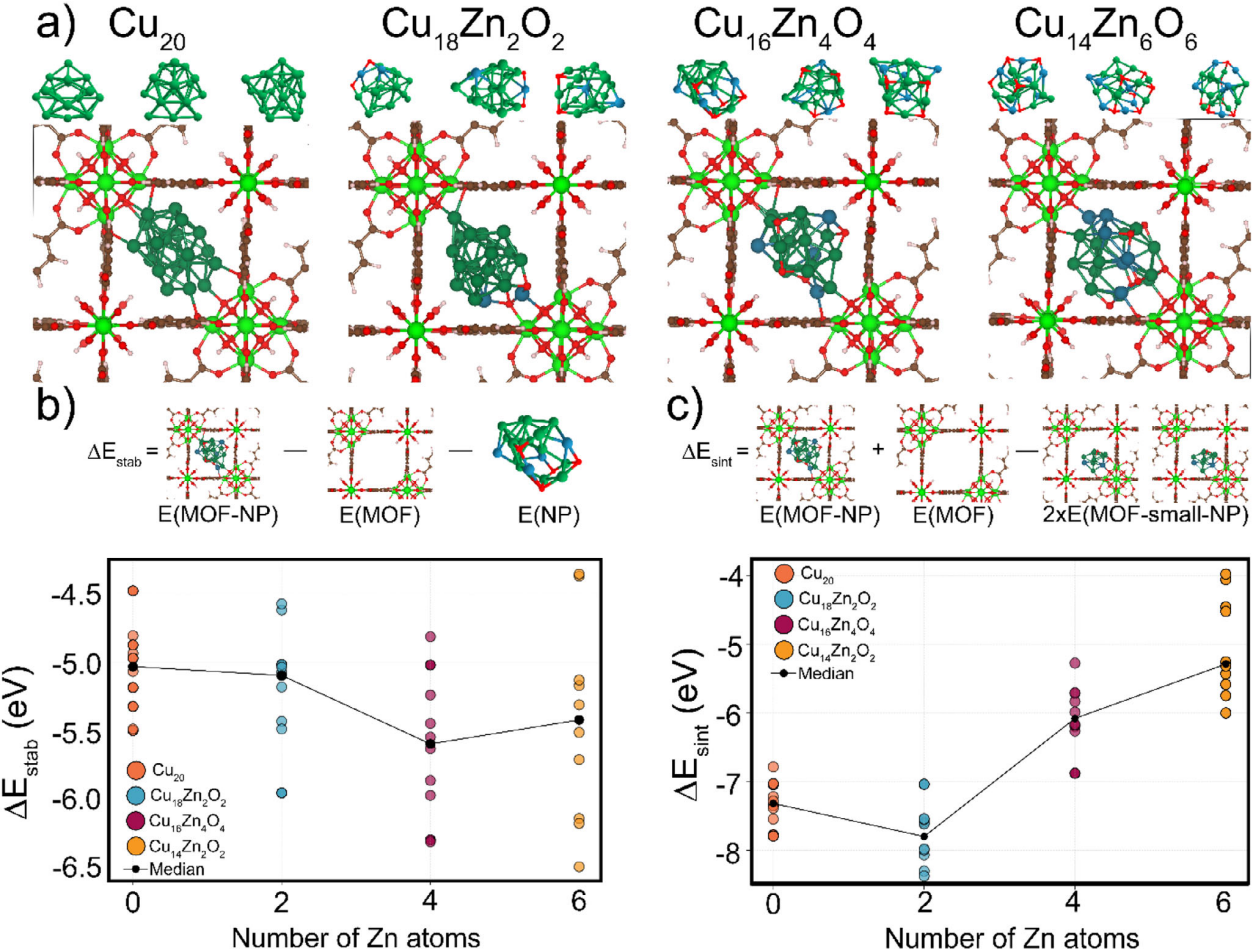
DFT analysis of Cu and Cu‐Zn nanoparticle stabilization in defective UiO‐66. (a) Representative lowest‐energy configurations of isolated and confined clusters of considered stoichiometries (b) Calculated stabilization energy and (c) sintering energies for the MOF‐confined nanoparticles with the increasing Zn content. Color code for atoms: H – white, C – brown, O – red, Cu – dark green, Zn – blue, Zr – light green.

Within this model framework, we investigated the confinement and stabilization of small Cu and Cu‐Zn clusters within the UiO‐66 framework, as summarized in Figure 9. To evaluate the stabilization of different nanoparticle species, we modeled various stoichiometries with increasing Zn composition: Cu_20_, Cu_18_Zn_2_O_2_, Cu_16_Zn_4_O_4_, and Cu_14_Zn_6_O_6_ clusters. The clusters were confined between two exposed Zr‐oxo node faces in missing‐linker defective UiO‐66; when accessible, the Zn‐rich face was oriented toward the defect to maximize Zn‐O_node_ interactions with the oxygenated node terminations. To ensure model reliability, we performed an exhaustive screening of the configurational space by generating 2000 + 125×n(Zn) isolated in vacuum structures for each composition using the PGOPT code [51], which were then optimized and ranked using the MACE‐MATPES‐PBE‐0 foundation model [52]. The top 10 most stable configurations for each composition were subsequently reoptimized with DFT to assess whether cluster topology significantly influences the stabilization trends.

Representative examples of isolated and confined particle geometries across different compositions are presented in Figure 9a. We first assessed particle stabilization in the MOF by calculating the stabilization energy (*ΔE_stab_*), which is the energy difference between MOF‐confined particles and isolated particles in the vacuum. The calculations reveal that while particle topology induces more scatter in individual stabilization energies, a clear systematic trend demonstrates that increasing Zn content shifts the stabilization energy distribution toward more negative values, indicating an increase in the amount stronger stabilized particles within the configurational ensemble (Figure 9b). This trend can be attributed to the significantly higher oxophilicity of Zn compared to Cu, which drives preferential binding of Zn‐rich faces to the oxygen‐terminated Zr‐oxo nodes at the defect sites [53].

To further investigate particle stabilization, we modeled a simplified sintering reaction (Figure 9c) in which two smaller confined clusters (Cu_10_, Cu_9_ZnO, Cu_8_Zn_2_O_2_, Cu_7_Zn_3_O_3_) combine into a larger aggregate, leaving one defect site vacant. The resulting sintering energy (*ΔE_sint_*) quantifies the thermodynamic driving force for particles to coalesce within the framework, with more negative values indicating a stronger tendency toward sintering. The results show that while all compositions exhibit a thermodynamic propensity toward sintering, the magnitude of this driving force depends strongly on cluster composition. While Cu_9_ZnO particles exhibit a slightly enhanced tendency toward sintering compared to Cu_10_, particles with higher Zn content demonstrate increased resistance to sintering. This trend indicates that Zn‐rich compositions benefit from enhanced MOF‐mediated stabilization against particle growth/sintering, consistent with the experimental observations discussed above, where Cu/UiO‐66 exhibits higher coordination numbers and a larger contribution of higher‐coordination metallic Cu compared to Cu/Zn‐UiO‐66.

Overall, the DFT results shown in Figure 9 support the experimental observation that UiO‐66 can stabilize small Cu‐based clusters and that incorporation of Zn further enhances this stabilization. These findings are consistent with the predominance of dispersed Cu^0^ species identified by Cu K‐edge XANES, the reduced coordination numbers derived from EXAFS, and the smaller Cu‐rich species observed by electron microscopy in Cu‐Zn/UiO‐66. Electron microscopy, operando XANES, EXAFS, and DFT calculations consistently demonstrate that Zn promotion alters Cu speciation within the UiO‐66 framework: in Cu/UiO‐66, Cu evolves toward a mixture of higher‐coordination and dispersed metallic species, whereas in Cu‐Zn/UiO‐66, Cu is predominantly stabilized as lower‐coordination dispersed species. This shift toward smaller and more dispersed Cu species suggests that the observed increase in methanol productivity may partly arise from a dispersion effect, in addition to stabilization under reaction conditions. At the same time, the present data do not allow identification of a unique methanol‐forming active site, and contributions from different Cu environments or Cu‐Zn interfacial motifs cannot be excluded. Further EXAFS analysis supports reduced Cu‐Cu coordination numbers compared to higher‐coordination metallic Cu, consistent with nanoscale Cu domains in Cu‐Zn/UiO‐66, suggesting that the presence of Zn contributes to the stabilization of these more dispersed Cu species. Operando XANES further shows that Zn does not exhibit detectable changes in its average local structure under the applied conditions, indicating a stable Zn environment. The Zr nodes of UiO‐66 respond dynamically through reversible hydroxylation and dehydroxylation during reaction and gas‐switching experiments, which is interpreted as a response to adsorbed species rather than direct evidence of catalytic involvement. Textural and spectroscopic characterization further demonstrates that the UiO‐66 framework remains structurally preserved upon Cu and Cu‐Zn species incorporation, reduction, and catalytic tests. N_2_ physisorption and Raman spectroscopy reveal changes in local structure and porosity upon precursor loading and reaction, indicating redistribution of introduced metallic species within the framework while preserving the overall framework structure. Subsequent transient catalytic experiments further show that the UiO‐66 matrix can adsorb and retain oxygenated species, indicating a dual function as catalyst support and adsorbent.

## 3 Conclusions

In this work, UiO‐66‐based catalysts promoted with Cu and Cu‐Zn were investigated for CO_2_ hydrogenation to methanol. Pristine UiO‐66 was shown to be catalytically inactive, while incorporation of Cu enabled significant methanol formation with high selectivity. Further promotion with Zn increased methanol productivity by ca. 15–20% at comparable Cu loading, while maintaining methanol selectivity close to 90% (1.8 mmol _MeOH_ g_cat_^−1^ h^−1^ for Cu/UiO‐66 and 2.1 mmol _MeOH_ g_cat_^−1^ h^−1^ for Cu‐Zn/UiO‐66).

The combined operando spectroscopic, microscopic, and computational results show that methanol productivity correlates with the presence of dispersed Cu species within the UiO‐66 framework, which are further stabilized in the presence of Zn under reaction conditions, consistent with a structural promotion effect rather than a directly observed active Zn phase. This stabilization persists under reaction and transient conditions. These observations suggest that the UiO‐66 framework contributes to the stabilization of inorganic species and the adsorption/retention of hydrogenated species during reaction. Incorporation of Cu enables methanol formation, while Zn promotion enhances productivity and brings changes in reactive states during the feed switches. The ability of the MOF to retain reaction intermediates and products under transient conditions indicates that the framework contributes beyond acting as a passive support.

These findings illustrate how a MOF host can be used to control metal speciation under working conditions and provide guidance for the design of MOF‐based catalysts for CO_2_ hydrogenation.

## 4 Experimental Section

UiO‐66 was synthesized by a solvothermal method [30]. Zirconium(IV) chloride (ZrCl_4_, 99.5%, Sigma–Aldrich) and terephthalic acid (1,4‐benzenedicarboxylic acid, 99%, Sigma–Aldrich) were dissolved in N,N‐dimethylformamide (DMF, anhydrous, 99.8%, Sigma–Aldrich), followed by the addition of aqueous HCl (32 wt.%, Sigma–Aldrich) as a modulator. The reaction mixture was heated in a sealed autoclave at 220°C for 20 h to yield UiO‐66. The resulting solid was recovered by centrifugation, washed repeatedly with DMF, and subjected to solvent exchange with methanol (HPLC grade, 99.8%, VWR). Final activation was performed under dynamic vacuum at elevated temperature to remove residual solvents.

Cu/UiO‐66 and Cu‐Zn/UiO‐66 were prepared by wet impregnation of pre‐synthesized and activated UiO‐66 using metal acetylacetonate precursors. UiO‐66 was suspended in dry acetonitrile (anhydrous, Sigma–Aldrich) under inert atmosphere, and appropriate amounts of copper(II) acetylacetonate (Cu(acac)_2_, ≥ 97%, Sigma–Aldrich) or a mixture of Cu(acac)_2_ and zinc(II) acetylacetonate (Zn(acac)_2_, ≥ 98%, Sigma–Aldrich) were added to obtain nominal metal loadings of approximately 4 wt.% Cu and 10 wt.% Zn. The suspensions were stirred overnight at room temperature, followed by solvent removal. The impregnated materials were thermally treated under vacuum at 150°C to partially decompose the precursors and anchor the metal species within the UiO‐66 framework. The resulting catalysts were stored under an inert atmosphere prior to characterization and catalytic testing.

^1^H NMR Spectroscopy: For sample preparation 5–10 mg of the MOF were dissolved by sonication in an aqueous ammonium bicarbonate solution 1 M prepared in D_2_O. ^1^H NMR spectra were recorded on a Magritek Spinsolve 60 MHz spectrometer at room temperature. Solvent signal suppression was applied using 50 dB presaturation, with 8192 scans and a 7 s repetition delay. Chemical shifts were referenced to the D_2_O signal.

Powder X‐ray diffraction (PXRD) patterns were collected using a Bruker D8 Advance diffractometer equipped with Cu Kα radiation (λ = 1.5406 Å). Measurements were performed at room temperature under ambient (air‐exposed) conditions. Samples were transferred from a glovebox prior to measurement and mounted on zero‐background sample holders. Data were collected over a 2θ range of 2°–80° with a step size of 0.04°. High‐energy synchrotron XRD measurements were performed at ID31, ESRF (Grenoble, France), using a photon wavelength of 0.0165 nm (75 keV) [54].

For TEM sample preparation, either continuous or lacey carbon grids with copper or molybdenum support (product numbers: S166‐3 from Plano and Mo‐400CN from Pacific Grid Tech) were used. The sample was first crushed using a mortar inside a glovebox, then dispersed in ethanol or methanol. The optimal sample concentration was achieved by varying the relative amounts of solvent and sample. Approximately 4 µL of the resulting suspension was deposited onto the grid and allowed to dry on filter paper. TEM and STEM‐EDX images were obtained using a probe‐corrected JEOL JEM‐ARM200F electron microscope operating at 200 keV acceleration voltage and equipped with cFEG. For the high‐resolution TEM, we used low dose mode for minimal damage of the MOFs. In STEM mode, using a cFEG, we used emissions between 5 and 15 µA, which corresponded to beam currents of 4–10 pA. For EDX‐based data acquisition, higher probe currents were used with two JEOL detectors. Data analysis and figure preparation were performed using the Analysis Station software (JEOL).

TGA‐MS was performed using a Mettler Toledo TGA/DSC 1 instrument. The amount of the MOF (5–25 mg) was placed in an alumina crucible and then heated up to 800°C with a ramp rate of 5°C min^−1^ in artificial air flow, 20 mL min^−1^. The TGA chamber outlet was connected to a mass spectrometer (MS, Pfeiffer Thermostar).

Chemical composition was measured by ICP‐OES at Agilent ICP‐OES 5110 spectrometer. Samples (5–20 mg) were digested using a mixture of concentrated acids (HNO_3_, 65 wt.%; HF, 40 wt.%; HCl, 37 wt.%) under microwave heating. Then we diluted them 10 times with mQ water and transferred to tubes for the measurement.

N_2_ Physisorption Measurements: The materials were activated overnight at 0.20 mbar and 150°C, using a Micromeritics VacPrep 061 sample preparation device. The weight loss due to activation was considered for the calculation of molar amounts of materials, and the N_2_ physisorption experiments were conducted on a Micromeritics 3Flex instrument. The average pore diameter was taken as the adsorption average pore diameter (4V/A by BET).

Raman spectra of UiO‐66 were recorded using a RamanTouch confocal Raman microscope (Bruker) equipped with a 532 nm excitation laser. The laser power at the sample was 0.51 mW. Spectra were collected using a 300 gr mm^−1^ grating, a 50 µm slit, and a 20× objective (NA = 0.45). Each spectrum was acquired with an integration time of 3 s and averaged over 20 accumulations. The CCD detector was operated in low‐noise mode and cooled to −70°C. Measurements were performed in point mode under ambient (air‐exposed) conditions. Data acquisition and processing were carried out using RAMAN Imager2 software.

The CO_2_ hydrogenation experiments in the gas phase were carried out using a fixed‐bed reactor with a feed of CO_2_, H_2_, N_2_. In a typical experiment, a stainless‐steel reactor with I.D. of 2.5 mm was charged with 50 mg of the UiO‐66‐based material + 100 mg SiC. The reactor was sealed with the metal frits with a pore size of 0.5 µm. Prior to catalysis, the catalyst was activated in an H_2_‐containing atmosphere (10 vol.% H_2_ in N_2_) at 220°C (heating rate 10°C min^−1^) and 5 bars for 1 h to remove possible organic contaminants and to reduce the introduced Cu and Cu‐Zn species. After the activation, the temperature was lowered to 200°C in pretreatment gas mix, and the system was pressurized to 20 bar. Then we kept the sample for ca. 2 h on CO_2_/H_2_ stream, then alternated the flow for 20 min first inert/ CO_2_, then 20 min of the reaction mix, then inert/H_2_ followed by the switch back to CO_2_/H_2_ for 20 min and then cooled down in the reaction mixture. Each test was carried out three times.

Online analysis of the CO_2_ hydrogenation experiments was performed by a gas chromatograph (GC, CompactGC 4.0, Global Analyzer Solutions) with heated gas transfer lines. The GC had four pre‐columns, four columns, three detectors, and a Methanizer device. A thermal conductivity detector (TCD) coupled with an RT‐Q‐Bond pre‐column (3 m; i.d. 0.32 mm; film thickness 10 µm) and a Molsieve 5A FS column (15 m; i.d. 0.32 mm; film thickness 30 µm) was used for the analysis of the permanent gases, including H_2_, CO_2_, N_2_ were analyzed by another TCD coupled with an RT‐Q‐Bond pre‐column (3 m; i.d. 0.32 mm; film thickness 10 µm) and an RT‐Q‐Bond column (12 m; i.d. 0.32 mm; film thickness 10 µm). Oxygenates and hydrocarbons were traced using an MXT‐5 column (12 m; i.d. 0.28 mm; film thickness 1 µm) coupled with MXT‐5 pre‐column (3 m; i.d. 0.28 mm; film thickness 1 µm) and analyzed with a flame ionization detector (FID). A separate channel connected to a Methanizer device and later to the FID detector was used for low CO concentrations. The channel was equipped with a Carboxen 1010 pre‐column (3 m; i.d. 0.32 mm) and Carboxen 1010 column (12 m; i.d. 0.32 mm). The selectivities and yields for the reaction were estimated from the peak areas observed at FID and calibrated with analytical mixture containing small concentrations of CO, methanol, methane, and ethylene, and using formulas provided here [23]. The response factor for methyl formate was estimated from here [55, 56].

The ex situ and operando characterization of the Cu, Zn species and Zr nodes in the UiO‐66, their local environment, and the chemical state was carried out using XAS for the measurements at ROCK beamline, SOLEIL [57]. The storage ring operated in multibunch mode at 500 ampere maximum current. Measurements were performed in transmission at Cu‐Zn simultaneously and Zr K‐edge, using 2 Si(111) channel‐cut monochromators aligned at 11.92° and 6.08°, respectively. The oscillation amplitude of the monochromator was set to 2.7° for Cu‐Zn K‐edges (covering an energy range from 8750 to 10 800 eV) and to 0.6° for the Zr K‐edge (covering an energy range from 17 800 to 19 200 eV). Harmonic rejection was ensured by mirrors aligned at 2.8 mrad with respect to the X‐ray beam before and after the monochromator, coated with B4C and Pd for Cu‐Zn and Zr K‐edge, respectively. The monochromator oscillates at 2 Hz, leading to an acquisition time for one XAS spectrum of 0.5 s in the ascending angles of the monochromator. To improve signal‐to‐noise ratio, consecutive spectra were further averaged leading to a time resolution of 20 s per spectrum. During operando experiments, spectra were collected alternatively every 30 s at Cu‐Zn and Zr K‐edges with 30 s to change from one edge to the other, taking advantage of the so‐called “edge‐jumping” capability offered by the ROCK beamline. Measurements were performed in transmission mode using ionization chambers (OKEN) filled at 1 bar with N_2_ for Cu‐Zn K‐edges and with a mixture of 50/50 N_2_/Ar for Zr K‐edge. The spectra of the Cu and Zr reference foil placed between I1 and I2 were measured together with the sample and then used for energy alignment. Spectra extraction, energy alignment, and normalization were performed with the Python GUIs scripts developed at the ROCK beamline [44]. EXAFS Fourier Transform was performed in the 3–12 Å^−1^ k‐range with Hanning window. EXAFS data processing and fitting were carried out using Athena and Artemis from the Demeter software suite [58]. For the fitting, 10 scans at the end of the first CO_2_/H_2_ exposure were averaged.

The operando catalytic hydrogenation of CO_2_ was carried out using the reaction system available in‐house and the gas rig provided by Operando Group, SLS, PSI. The setup allows control over reactant composition, flow rates, pressure, and temperature under reaction conditions compatible with operando XAS [59]. The gas feed lines were connected to high‐purity CO_2_, H_2_, and Ar cylinders. The gas flow was distributed to the setup with a custom‐built setup, which consists of a series of Bronkhorst mass flow controllers (MFCs), each dedicated to individual gases involved in the experiment (H_2_, CO_2_, Ar), a fast‐switching VICI valve, and a back‐pressure regulator (BPR, Bronkhorst). The fast‐switching valve enabled us to switch from one flow condition to another with minimized delay, even at high pressure. The scheme for the gas flow setup is provided in Figures S13 and S14. For the operando experiments, 5–15 mg of the powder sample was packed between two quartz wool plugs into a 2 mm diameter, 0.1 mm thick‐walled quartz capillary to form an 8 mm long catalyst bed. The capillary was then sealed in a U‐shaped frame using epoxy or UV‐active adhesive and attached with Swagelok connections to a capillary reactor setup developed by the ROCK beamline at SOLEIL. The capillary was aligned in the X‐ray beam path. The reactor was heated with a gas blower; the outlet was connected to a Bronkhorst back‐pressure regulator, and the gas composition was continuously monitored with a mass spectrometer during the XAS operando experiment. The heating assembly was calibrated in the range of 50–250°C under Ar flow. For the operando CO_2_ hydrogenation experiments, we used the edge jumping feature of the ROCK beamline. These experiments were designed to reproduce the catalytic testing protocol. An initial state spectrum was recorded for the sample at both edges under Ar flow (30 mL min^−1^) at RT. This was followed by operando pre‐treatment in a 10%H_2_/Ar mixture at 220°C, 5 bar, for 1 h with a 5°C min^−1^ ramp. After the pretreatment, the reactor was first cooled to reaction temperature (200°C), pressurized to 20 bar in the same gas mixture (10%H_2_/Ar, 30 mL min^−1^), and then switched to the reaction mixture to study the transient effect of the introduction of reactants to the Cu, Zn, and Zr species. The cell outlet was connected to an exhaust and a mass spectrometer (Pfeiffer Omnistar). Data pre‐processing was performed using the Python GUIs developed at the ROCK beamline (namely ‘extract_gui’ and ‘normal_gui’) [44].

Spectra energy alignment, background subtraction, edge jump normalization, and EXAFS extraction were performed with Larch‐based python scripts [60]. Python‐based tools were developed and used in this work. In XAS measurements of multicomponent systems, each spectrum can be expressed as a linear combination of pure component spectra weighted by their relative contributions [61]. Accordingly, a series of spectra collected under varying experimental conditions (e.g., temperature or gas composition) can be represented as a bilinear matrix decomposition [62]. The number of components describing a dataset was first estimated using Principal Component Analysis (PCA) [62, 63]. The PCA was performed using Python scripts based on the Pyfitit library [64]. Multivariate Curve Resolution‐Alternating Least Squares (MCR‐ALS) method was applied to resolve spectral components and their evolution [65, 66]. MCR‐ALS was performed using Python scripts based on pymcr library [67]. This method enables blind decomposition without prior reference spectra and is well suited for exploratory analysis of evolving catalytic systems. Initial estimates for MCR‐ALS were generated using the SIMPLISMA algorithm [68]. The overall workflow consisted of PCA to determine the number of significant components, followed by MCR‐ALS to extract concentration profiles and spectral components using a mixture of initial guess spectrum generated by SIMPLISMA and known references measured in the same XAS experiment. Principal component analysis results and corresponding residuals for Cu and Zr K‐edge XANES of Cu/UiO‐66 and Cu‐Zn/UiO‐66 are provided in Figures S19 and S24. For the datasets measured at Cu K‐edge and Zr K‐edge, MCR‐ALS analysis was performed on the combined datasets of Cu/UiO‐66 and Cu‐Zn/UiO‐66. At Cu K‐edge, four components were used in the iteration: (i) Cu foil (imported reference, fixed), (ii) Cu(acac)_2_ (imported reference, fixed, corresponding to precursor species), (iii) a CuO‐like component (initialized from SIMPLISMA and allowed to vary during iteration), and (iv) a lower‐coordination Cu‐species component (initialized from SIMPLISMA and allowed to vary). At Zr K‐edge, two SIMPLISMA‐generated components were used as initial guess and allowed to vary during iteration. Additional parameters affecting the MCR‐ALS results are discussed in Section S6.

## Details for DFT Calculations

**Methods**: All DFT calculations were performed using the Vienna Ab Initio Simulation Package (VASP 6.4.3) [69]. The exchange‐correlation energy was described with the PBE functional and D3BJ dispersion corrections [70, 71, 72]. A plane‐wave basis set with a cutoff of 550 eV in combination with the projected augmented wave (PAW) method [73] was applied. Electronic self‐consistency was converged to an energy tolerance of 10^−5 ^eV. Gaussian smearing with a width of 0.05 eV was applied to partial occupancies during the iterative diagonalization of the KS Hamiltonian. Geometries were considered converged when all the forces acting on the atoms were below 0.04 eV Å^−1^. Brillouin zone sampling was restricted to the Γ‐point.

Models:

- **Nanoparticles structure generation**. To ensure representative nanoparticle models, we performed an exhaustive search of gas‐phase nanoparticle configurations. Initial nanoparticle geometries were generated using the PGOPT code [14, 51], with the number of structures equal to 2000 + 125 × n(Zn), where n(Zn) is the number of Zn atoms in the nanoparticle (e.g., 2000 for Cu_20_, 2500 for Cu_18_Zn_2_O_2_, etc.), with the exception of Cu_14_Zn_6_O_6_, for which 4500 models were constructed. These structures were inserted in the center of 20 × 20 × 20 Å periodic box, optimized, and ranked using the MACE‐MATPES‐PBE‐0 [52] foundation model. The model was used as‐is, without further fine‐tuning. The performance of this model was validated against DFT data Figure S36, demonstrating good agreement in ranking stable and unstable nanoparticle configurations. The configurational space coverage is illustrated in Figure S37, confirming adequate sampling of the potential energy surface.
- **MOF model construction**. The cubic UiO‐66‐unit cell was used as the starting structure. A defect site was created by removing one terephthalate dianionic linker (C_8_H_4_(COO)_2_)^2−^ and deprotonating two MOF nodes exposed after the linker removing. The unit cell was optimized for both ionic positions and cell parameters (with the cell shape fixed), yielding lattice parameters of *a* = *b* = *c* = 20.87 Å. Nanoparticles were then inserted between the exposed nodes, and if possible, a Zn‐rich edges were attached to the nodes. For sintering energy calculations, only the lowest‐energy small nanoparticle configuration from each composition was used.

## Author Contributions

**Anna Liutkova**: conceptualization, writing – original draft, writing – review and editing, investigation, data curation, methodology, validation, and visualization. **Fabio André Peixoto Esteves**: methodology, writing – review, and editing. **Marianela López Romero**: methodology, investigation, writing – review, editing, and data curation. **Margareth S. Baidun**: conceptualization, investigation, methodology, software, writing – review, editing, and data curation. **Anastasia Molokova**: investigation, methodology, software, writing – review, editing, data curation, and formal analysis. **Davide Salusso**: software, formal analysis, Writing – review, and editing. **Kirill A. Lomachenko**: methodology, writing – review, editing, software, and formal analysis. **Andrea Testino**: writing – review, editing, and formal analysis. **Alexander A. Kolganov**: conceptualization, investigation, methodology, software, writing – review, editing, and data curation. **Evgeny A. Pidko**: writing – review, editing, methodology, conceptualization, computational resources, and funding acquisition. **Emiliya Poghosyan**: writing – review, editing, methodology, formal analysis, and investigation. **Marco Ranocchiari**: conceptualization, funding acquisition, writing – original draft, writing – review, editing, supervision, resources, and project administration.

## Conflicts of Interest

The authors declare no conflicts of interest.

## Supporting information




**Supporting File**: smll74210‐sup‐0001‐SuppMat.docx.

## Data Availability

The data that support the findings of this study are openly available in ESRF at https://doi.esrf.fr/10.15151/ESRF‐ES‐2140750475.
