# Supplementary material for: Stabilization of Cu Species in UiO‐66 Metal–Organic Framework for CO2‐to‐Methanol: Insights From Operando X‐ray and Electron Microscopy Studies
Source: Small. 2026 Jul 1;22(48):e74210. doi: 10.1002/smll.74210 (PMC13509059; doi:10.1002/smll.74210)
Supplement: Supplementary file 1 [file SMLL-22-e74210-s001.docx]

**Supporting information**

to

Stabilization of Cu Species in UiO-66 Metal-Organic Framework for CO_2_-to-Methanol: Insights from Operando X-ray and Electron Microscopy Studies

Anna Liutkova^1*^, Fabio André Peixoto Esteves^1,2^, Marianela López Romero^1,3^, Margareth S. Baidun^4^, Anastasia Molokova^3,5^, Davide Salusso^3^, Kirill A. Lomachenko^3^, Andrea Testino^1,6^, Alexander A. Kolganov^4^, Evgeny A. Pidko^4^, Emiliya Poghosyan^7^ and Marco Ranocchiari^1*^

*^1^ Center for Energy and Environmental Sciences, PSI, Forschungsstrasse 111, 5232 Villigen PSI, Switzerland*

*^2^ Department of Chemistry and Applied Biosciences, ETH Zürich, Vladimir Prelog Weg 1-5, 8093 Zurich, Switzerland*

*^3^ European Synchrotron Radiation Facility, 71 Avenue des Martyrs, 38000 Grenoble, France*

*^4^ Inorganic Systems Engineering group, Department of Chemical Engineering, Faculty of Applied Sciences, Delft University of Technology, Delft, the Netherlands*

*^5^ ALBA synchrotron, Carrer de la Llum, 2, 26, 08290 Cerdanyola del Vallès, Barcelona, Spain*

*^6^ École Polytechnique Fédérale de Lausanne (EPFL), Lausanne 1015, Switzerland*

*^7^ Center for Life Sciences, PSI, Forschungsstrasse 111, 5232 Villigen PSI, Switzerland*

Corresponding authors:

Anna Liutkova

Center for Energy and Environmental Sciences

Paul Scherrer Institute PSI

Forschungsstrasse 111

5232 Villigen PSI, Switzerland

Marco Ranocchiari

Center for Energy and Environmental Sciences

Paul Scherrer Institute PSI

Forschungsstrasse 111

5232 Villigen PSI, Switzerland

**Contents**

[1.	Synthesis and bulk characterization	2](#_Toc230026044)

[2.	Electron microscopy: pristine and fresh materials	4](#_Toc230026045)

[3.	An overview of UiO-66-based Cu catalysts and reported performance metrics	8](#_Toc230026046)

[4.	Structural stability and framework response	9](#_Toc230026047)

[5.	Operando XAS setup and gas analysis	11](#_Toc230026048)

[6.	XANES reference data and multivariate analysis	13](#_Toc230026049)

[6.1	Additional parameters affecting MCR-ALS results	17](#_Toc230026050)

[7.	EXAFS analysis	18](#_Toc230026051)

[8.	Details of the EXAFS fitting	21](#_Toc230026052)

[8.1	Cu K-edge	21](#_Toc230026053)

[8.2	Zn K-edge	22](#_Toc230026054)

[9.	Nanoparticle model generation and validation	24](#_Toc230026055)

[References	25](#_Toc230026056)

## 1. Synthesis and bulk characterization

**Figure S1.** Synthesis procedure for the Cu/UiO-66 and Cu-Zn/UiO-66 catalysts.

**Figure S2.** (a,b) XRD of UiO-66, fresh Cu/UiO-66, and fresh Cu-Zn/UiO-66. The areas highlighted with light orange, light grey, and light blue correspond to possible phases from Cu, CuO, and ZnO. (c) ^1^H NMR spectra of freshly prepared UiO-66, Cu/UiO-66, and Cu-Zn/UiO-66.

## 2. Electron microscopy: pristine and fresh materials

**Figure S3.** TEM micrograph-based overview of the pristine UiO-66.

**Figure S4.** TEM (a, c, d) and HAADF STEM-EDX micrographs of fresh Cu/UiO-66 (a,b,e,f) and fresh (c,d,g,h) Cu-Zn/UiO-66 showing the distribution of Cu and Zn species.

**Figure S5.** HAADF-STEM micrographs and line scan distribution for the ZnO species in the used Cu-Zn/UiO-66 (a,c), and Cu particles in the reduced Cu/UiO-66 sample, adapted from **Figure 4c**, (b,d), both species exhibiting a crystalline structure. (e, f) features, where panel (f) shows zoomed in region from panel (e), indicated by black rectangle.

**Figure S6.** STEM-EDX elemental maps of a Zn-containing domain in Cu-Zn/UiO-66 showing the spatial distribution of Zn (a), C (b), Mo (c), O (d), and Cu (f), together with the corresponding EDX spectrum (e). Co-localization of Zn, Cu, and O signals supports the presence of Zn-containing oxide domains spatially associated with Cu-rich species.

**Figure S7.** HAADF STEM micrographs of reduced Cu/UiO-66 showing small bright nanoparticles decorating the MOFs.

**Figure S8.** HAADF STEM micrographs of reduced Cu-Zn/UiO-66 showing small bright nanoparticles decorating the MOFs.

**Figure S9.** HAADF STEM micrographs of used Cu-Zn/UiO-66 showing small bright nanoparticles decorating the MOFs.

## 3. An overview of UiO-66-based Cu catalysts and reported performance metrics

**Table S1.** Comparison of some UiO-66-based Cu catalysts reported for CO_2_ hydrogenation to methanol.

| UiO-66-based Cu catalyst  (as named in the paper) | *T* [°C] | *P* [bar] | *H_2_/CO_2_* | *GHSV* [h^-1^] | *Cu* [wt%] | *S_BET_* [m^2^ g^-1^] | *V_pore, total_* [cm^3^ g^-1^] | MeOH productivity [mmol g_cat_^-1^ h^-1^],  otherwise stated | Source |
| --- | --- | --- | --- | --- | --- | --- | --- | --- | --- |
| Cu/UiO-66 | 200 | 20 | 3 | 36 000 | 4.1 | 940 | 0.78 | 1.8 or 44 mmol g_Cu_^-1^ h^-1^ | This work |
| Cu-Zn/UiO-66 | 200 | 20 | 3 | 36 000 | 4.1 (Cu); 10.2 (Zn) | 880 | 0.64 | 2.1 or 51 mmol g_Cu_^-1^ h^-1^ | This work |
| Cu/UiO-66-a | 190 | 20 | 3 | 24 000 | 1.4 | 1468** | 0.61 | 1.0 (converted from 4.7 mol mol_Cu_ h^-1^) | ^[1]^ |
| Cu/UiO-66-b | 190 | 20 | 3 | 24 000 | 1.8 | 1432** | 0.60 | 0.02 (converted from 0.1 mol mol_Cu_ h^-1^) |  |
| Cu/UiO-66-a-high | 190 | 20 | 3 | 24 000 | 7.6 | NR | NR | 1.6 (converted from 1.3 mol mol_Cu_ h^-1^) |  |
| 15-CZ@U-2.5 | 240 | 30 | 3 | 2 400 | 10.5 | 155 | 0.18 | 0.7 | ^[2]^ |
| 35-CZ@U-2.5 | 240 | 30 | 3 | 12,000 | 22.9 | 96 | 0.14 | 6.4 |  |
| Cu⊂UiO-66 | 175 | 10 | 3 | NR | 1.0 | 954 | NR | TOF = 3.7 × 10^-3^ s^-1^ | ^[3]^ |
| Cu on UiO-66 | 175 | 10 | 3 | NR | 1.4 | 1040 | NR | TOF = 3.7 × 10^-3^ s^-1^ |  |
| Cu@UiO-66 | 220 | 10 | 3 | 21 600 | 5.8 | 807 | 0.20 | 3.0 (converted from 1.7 g_MeOH_ g_Cu_^-1^ h^-1^) | ^[4]^ |
| Cu/UiO-66(Zr) | 200 | 18 | 3 | NR | 3.5 | 757 | 0.51 | 0.01 mmol g_cat_^-1^ h^-1^  (converted from 397 µmol_MeOH_ g_Cu_^-1^ h^-1^ ) | ^[5]^ |
| CuZn@UiO-bpy | 250 | 40 | 3 | 18 000 | 6.9 | 118 | NR | 5.6 mmol g_cat_^-1^ h^-1^  (converted from 2.6 g_MeOH_ g_Cu_^-1^ h^-1^ ) | ^[6]^ |
| Cu/ZnO_x_@UiO-66 | 250 | 40 | 3 | 12 000 | 5.9 | 64 | NR | 2.3 mmol g_cat_^-1^ h^-1^  (converted from 1.3 kg _MeOH_ kg_Cu_^-1^ h^-1^) | ^[7]^ |
| Cu@UiO-66 | 250 | 40 | 3 | 12 000 | 6.7 | 262 | NR | 0.8 mmol g_cat_^-1^ h^-1^  (converted from 0.4 kg _MeOH_ kg_Cu_^-1^ h^-1^) |  |

* NR - not reported; ** - specific surface area

While **Table S1** compares UiO-66-based Cu catalysts using commonly reported metrics, this comparison is not exhaustive. As highlighted in a recent critical review^[8]^, catalytic performance in Zr_6_-MOF-based systems is strongly influenced by additional parameters that are not consistently reported across studies, including Cu particle size and location (encapsulated vs. external), density and type of framework defects (missing linker and missing cluster defects), extent of Cu-ZrO_2_ interfacial sites, presence and distribution of promoters such as Zn, and the evolution of metal speciation under reaction conditions. Moreover, differences in synthesis route, reduction history, water tolerance, and reaction pressure and space velocity can significantly affect apparent activity and selectivity.

## 4. Structural stability and framework response

**Figure S10.** XRD of fresh and used catalysts compared. (a,b,d) measured at ID31, ESRF. Conditions at ID31, ESRF: samples are measured in a capillary cell, quartz capillary 1.5 mm thickness, 15 bar, CO_2_:H_2_ 1:3, 200 °C reaction, 30 mL/min, q = 0.3 - 6 Å^−1^ converted to Cu K Alpha 1.54 Å, 75 keV. (c,e) – powder XRD, measured at Cu Kα 1.54 Å lab source. The areas highlighted with light orange, light grey, and light blue correspond to possible phases from Cu, CuO, and ZnO.

**Figure S11.** Ex situ Raman of the pristine UiO-66, fresh and used catalysts. Conditions: excitation wavelength 532 nm, green; spectra are background-corrected.

**Figure S12.** (a) TGA of the pristine UiO-66 materials from 2 different batches and after CO_2_ hydrogenation catalytic test. (b) Subsequent MS measurements of the UiO-66 batch 1. Conditions: 50-800 °C, 5 °C/min, air, 20 mL/min flow.

## 5. Operando XAS setup and gas analysis

**Figure S13.** Image of the stage of the experiment and the XAS setup.

**Figure S14.** Custom-built gas flow setup enabling the fast-switching between two distinct gas streams (arriving from positions A and B) provided by Operando group and SuperXAS X10DA beamline, PSI.^[9]^

**Figure S15.** MS spectra of the chosen masses for the operando XAS experiment over UiO-66 (a), Cu/UiO-66 (b), Cu-Zn/UiO-66 (c); m/z 2 corresponds to hydrogen, 31 to methanol, 40 to Ar, and 44 to CO_2_.

## 6. XANES reference data and multivariate analysis

**Figure S16.** Operando XANES at the ROCK beamline (SOLEIL) during CO_2_ hydrogenation: Cu K-edge MCR-ALS concentration profile for Cu/UiO-66 of the lower-coordination Cu species component, evolution of the lower-coordination Cu species upon the switches. The assignment of components is supported by comparison to reference spectra and literature, but is not considered uniquely size-specific.

**Figure S17.** Cu, Zn, Zr K edge XAS spectra of the reference compounds.

**Figure S18.** Cu K edge XANES over Cu/UiO-66 (a-c), Cu-Zn/UiO-66 (d-f) at various stages of the experiment: activation, reaction + switches, cooldown.

**Figure S19.** Principal component analysis and residuals for Cu K-edge XANES for Cu/UiO-66 and Cu-Zn/UiO-66 samples.

**Figure S20.** Zn K edge XANES over Cu-Zn/UiO-66 (a-c) at various stages of the experiment: activation, reaction + switches, cooldown.

**Figure S21.** Zn K edge XANES over Cu-Zn/UiO-66 at various stages of experiment: RT- room temperature before any treatment, before reaction at 200 °C, after reaction at 200 °C (used), compared to a spectrum of a reference ZnO.

**Figure S22.** Zr K-edge XANES for Cu/UiO-66 collected operando at ROCK (SOLEIL) during CO_2_ hydrogenation at 200 °C, 20 bar, 30 mL min^-1^, CO_2_:H_2_ = 1:3, showing MCR-ALS components assigned as hydroxylated and dehydroxylated Zr species.

**Figure S23.** Zr K edge XANES over UiO-66 (a-c), Cu/UiO-66 (d-f), Cu-Zn/UiO-66 (g-i) at various stages of the experiment: activation, reaction + switches, cooldown.

**Figure S24.** Principal component analysis and residuals for Zr K-edge XANES for UiO-66-based samples collected operando at ROCK (SOLEIL) during CO_2_ hydrogenation at 200 °C, 20 bar, 30 mL min^-1^, CO_2_:H_2_ = 1:3.

### 6.1 Additional parameters affecting MCR-ALS results

Several additional parameters influence the MCR-ALS results, including the permitted increase in the error attribute, the number of consecutive iterations during which the error is allowed to increase, the number of half-iterations that can be performed without reaching a new error minimum, and the convergence criterion based on the minimum change in error per iteration required to continue the optimization.

During the MCR analysis, all these parameters were tested to ensure that the solution was not trapped in a local minimum. This involved allowing higher temporary error increases and reducing the convergence threshold to values several orders of magnitude smaller than the final Mean Squared Error (MSE). The reported MCR solutions correspond to the concentration and spectral profiles obtained at the iteration with the minimum MSE, which was 3.43e-5.

**Figure S25.** Evolution of the mean squared error (MSE) during the MCR-ALS optimization procedure.

## 7. EXAFS analysis

**Figure S26.** Cu K edge EXAFS k space over Cu/UiO-66 (a-c), Cu-Zn/UiO-66 (d-f) at various stages of the experiment: activation, reaction + switches, cooldown.

**Figure S27.** Cu K edge EXAFS R space over Cu/UiO-66 (a-c), Cu-Zn/UiO-66 (d-f) at various stages of the experiment: activation, reaction + switches, cooldown.

**Figure S28.** Zn K edge EXAFS, k space over Cu-Zn/UiO-66 (a-c) at various stages of the experiment: activation, reaction + switches, cooldown.

**Figure S29.** Zn K edge EXAFS, R space over Cu-Zn/UiO-66 (a-c) at various stages of the experiment: activation, reaction + switches, cooldown.

**Figure S30.** Zr K edge EXAFS, k space over UiO-66 (a-c), Cu/UiO-66 (d-f), Cu-Zn/UiO-66 (g-i) at various stages of the experiment: activation, reaction + switches, cooldown.

**Figure S31.** Zr K edge EXAFS, R space over UiO-66 (a-c), Cu/UiO-66 (d-f), Cu-Zn/UiO-66 (g-i) at various stages of the experiment: activation, reaction + switches, cooldown.

## 8. Details of the EXAFS fitting

### 8.1 Cu K-edge

The amplitude reduction factor S_0_^2^ was first determined by fitting the EXAFS spectrum of a Cu metal foil, using a fixed coordination number N = 12 (**Figure S32**). This procedure yielded S_0_^2^ = 0.9. For the fits of Cu-Zn/UiO-66 and Cu/UiO-66, S_0_^2^ was fixed to this value and incorporated into the amplitude term as S_0_^2^*N_i_, where N_i_ is a scaling parameter refined during the fit. The effective coordination numbers N_av_ for the samples were subsequently obtained by multiplying the refined N_i_ values by 12, corresponding to the coordination number of higher-coordination metallic-fcc Cu. The extracted coordination numbers should be interpreted as averages over the Cu environments present in the samples and are discussed together with the XANES/MCR-ALS results. Because the same fitting model and parameters were used for both catalysts, the relative difference in Cu-Cu coordination is used as the main basis for comparison. For the EXAFS analysis, averaged spectra were used. Three spectra were averaged for the Cu foil fit, while ten spectra collected during the stable stage of the reaction were averaged for each sample fit.

**Figure S32.** Averaged EXAFS spectra and their fit of the Cu foil.

**Figure S33.** Experimental and best-fit curves of the imaginary part of the Cu K-edge EXAFS spectra in k-space for Cu foil, Cu/UiO-66, and Cu-Zn/UiO-66. The shaded area indicates the fitting window.

### 8.2 Zn K-edge

We performed an EXAFS fit at Zn K edge for the spectra collected in three moments of the experiment over Cu-Zn/UiO-66 sample:

1) Room temperature - the first scan, before starting the experiment in H_2_/inert;
2) 200 °C before reaction - averaged last 7 scans at 200 °C after the temperature in H_2_/inert;
3) 200 °C after reaction - averaged last 7 scans at 200 °C of the reaction stage of the experiment in CO_2_/H_2_ reaction mixture;

The fitting results are presented in **Table S2**.

The EXAFS spectra collected at room temperature and at 200 °C were well described using the ZnO structural model, confirming that the local ZnO coordination environment is retained under reaction conditions. The fitted Zn-O and Zn-Zn distances remain consistent with the ZnO model, while the Debye-Waller factors (σ^2^) increase at elevated temperature. This increase is attributed to enhanced thermal vibrations and dynamic disorder at 200 °C, with the larger change observed for the Zn-Zn shell reflecting the greater sensitivity of higher-shell scattering paths to lattice disorder and nanoparticle dynamics.

**Table S2.** Fit results of the averaged spectra of the Zn species in Cu-Zn/UiO-66 sample, recorded before the experiment at room temperature, before the reaction at 200 °C and after the reaction at 200 °C in Cu-Zn/UiO-66.

| Condition | Path | *N* | *S_0_^2^* | *σ^2^* [Å^2^] | *E_0_* [eV] | *R* [Å] | *R-factor* |
| --- | --- | --- | --- | --- | --- | --- | --- |
| Room temperature, fresh | Zn-O | 4 | 0.9 (fixed) | 0.006±0.001 | 2.7 | 1.96±0.01 | 0.02 |
|  | Zn-Zn | 12 |  | 0.0096±0.0008 |  | 3.23±0.01 |  |
| 200 °C before reaction | Zn-O | 4 | 0.9 (fixed) | 0.0069±0.0007 | 2.2 | 1.96±0.01 | 0.02 |
|  | Zn-Zn | 12 |  | 0.0149±0.0008 |  | 3.23±0.01 |  |
| 200 °C after reaction (used) | Zn-O | 4 | 0.9 (fixed) | 0.007±0.001 | 2.0 | 1.96±0.01 | 0.02 |
|  | Zn-Zn | 12 |  | 0.014±0.001 |  | 3.22±0.01 |  |

**Figure S34. |**FT| of averaged k^2^-weighted EXAFS spectra and their fit of the Zn species in Cu-Zn/UiO-66. The shaded area indicates the fitting window.

**Figure S35.** Experimental and best-fit curves of the imaginary part of the Zn K-edge EXAFS spectra in k-space for the Zn species in Cu-Zn/UiO-66. The shaded area indicates the fitting window.

## 9. Nanoparticle model generation and validation

**Figure S36.** Correlation between DFT energies and MACE-MP predicted energies for selected configurations from a pool of 2500 initial Cu_18_Zn_2_O_2_ structures. Data points include the top-20 most stable structures predicted by MACE-MP, together with additional structures sampled at progressively higher predicted energies (structures numbered 100, 200, 300, 400, 500, 750, 1000, 1250, 1500, 1750, 2000, 2250 in the MACE-MP energy-sorted list).

To validate the MACE-MP model used for prescreening the cluster configurations, we compared the MACE-MP predicted energies with DFT energies for a representative set of Cu_18_Zn_2_O_2_ structures sampling low- and higher-energy regions of the configurational space. **Figure S36** shows that the model succeeds at ranking stable and unstable nanoparticle configurations and can be used to rank candidates’ structures prior to DFT refinement. The DFT analysis presented here focuses on relative thermodynamic stabilization trends of Cu- and Cu-Zn-containing nanoparticles confined within UiO-66. Explicit kinetic migration barriers associated with particle coarsening and diffusion were not evaluated in the present work.

**Figure S37.** Energy distribution of nanoparticle configurations generated by PGOPT and optimized with MACE-MP foundation model. Histograms show the relative energy distributions for considered nanoparticles stoichiometries.

**Figure S38.** Radial distribution functions for Cu-Cu bonds in MOF-confined nanoparticles of different compositions.

## References

[1]	Y. Zhu, J. Zheng, J. Ye, Y. Cui, K. Koh, L. Kovarik, D. M. Camaioni, J. L. Fulton, D. G. Truhlar, M. Neurock, C. J. Cramer, O. Y. Gutiérrez, J. A. Lercher, *Nat. Commun.* **2020**, *11*, 5849.

[2]	J. Yu, G. Chen, Q. Guo, X. Guo, P. Da Costa, D. Mao, *Fuel* **2022**, *324*, 124694.

[3]	B. Rungtaweevoranit, J. Baek, J. R. Araujo, B. S. Archanjo, K. M. Choi, O. M. Yaghi, G. A. Somorjai, *Nano Lett.* **2016**, *16*, 7645.

[4]	M. Song, T. Liu, X. Hong, G. Liu, *ACS Sustain. Chem. Eng.* **2023**, *11*, 12135.

[5]	M. Stawowy, R. Ciesielski, T. Maniecki, K. Matus, R. Łużny, J. Trawczynski, J. Silvestre-Albero, A. Łamacz, *Catalysts* **2019**, *10*, 39.

[6]	B. An, J. Zhang, K. Cheng, P. Ji, C. Wang, W. Lin, *J. Am. Chem. Soc.* **2017**, *139*, 3834.

[7]	Y. Yang, Y. Xu, H. Ding, D. Yang, E. Cheng, Y. Hao, H. Wang, Y. Hong, Y. Su, Y. Wang, L. Peng, J. Li, *Catal. Sci. Technol.* **2021**, *11*, 4367.

[8]	E. Tezel, D. K. Sannes, S. Svelle, P. Á. Szilágyi, U. Olsbye, *Mater. Adv.* **2023**, *4*, 5479.

[9]	S. Phadke, Deciphering Structure- Activity Relationships in Pd-Zn Catalysts for CO2 Hydrogenation to Methanol through Advanced Synthesis and XAS Characterization, ETH Zurich, **2025**.
